# Delegation to artificial intelligence can increase dishonest behaviour

**DOI:** 10.1038/s41586-025-09505-x

**Published:** 2025-09-17

**Authors:** Nils Köbis, Zoe Rahwan, Raluca Rilla, Bramantyo Ibrahim Supriyatno, Clara Bersch, Tamer Ajaj, Jean-François Bonnefon, Iyad Rahwan

**Affiliations:** 1 Research Center Trustworthy Data Science and Security, University Duisburg-Essen, Duisburg, Germany; 2 Center for Humans and Machines, Max Planck Institute for Human Development, Berlin, Germany; 3 Center for Adaptive Rationality, Max Planck Institute for Human Development, Berlin, Germany; 4 Toulouse School of Economics, CNRS (TSM-R), University of Toulouse Capitole, Toulouse, France

**Keywords:** Economics, Decision making, Human behaviour

## Abstract

Although artificial intelligence enables productivity gains from delegating tasks to machines^1^, it may facilitate the delegation of unethical behaviour^2^. This risk is highly relevant amid the rapid rise of ‘agentic’ artificial intelligence systems^3,4^. Here we demonstrate this risk by having human principals instruct machine agents to perform tasks with incentives to cheat. Requests for cheating increased when principals could induce machine dishonesty without telling the machine precisely what to do, through supervised learning or high-level goal setting. These effects held whether delegation was voluntary or mandatory. We also examined delegation via natural language to large language models^5^. Although the cheating requests by principals were not always higher for machine agents than for human agents, compliance diverged sharply: machines were far more likely than human agents to carry out fully unethical instructions. This compliance could be curbed, but usually not eliminated, with the injection of prohibitive, task-specific guardrails. Our results highlight ethical risks in the context of increasingly accessible and powerful machine delegation, and suggest design and policy strategies to mitigate them.

## Abstract (web summary)

People cheat more when they delegate tasks to artificial intelligence, and large language models are more likely than humans to comply with unethical instructions—a risk that can be minimized by introducing prohibitive, task-specific guardrails.

## Main

People are increasingly delegating tasks to software systems powered by artificial intelligence (AI), a phenomenon we call ‘machine delegation’^6,7^. For example, human principals are already letting machine agents decide how to drive^8^, where to invest their money^9,10^ and whom to hire or fire^11^, as well as how to interrogate suspects and engage with military targets^12,13^. Machine delegation promises to increase productivity^14,15^ and decision quality^16–18^. One potential risk, however, is that it will lead to an increase in ethical transgressions, such as lying and cheating for profit^2,19,20^. For example, ride-sharing algorithms tasked with maximizing profit urged drivers to relocate to artificially create surge pricing^21^; a rental pricing algorithm marketed as ‘driving every possible opportunity to increase price’ engaged in unlawful price fixing^22^; and a content-generation tool claiming to help consumers write compelling reviews was sanctioned for producing false but specific claims based on vague generic guidance from the user^23^. In this article, we consider how machine delegation may increase dishonest behaviour by decreasing its moral cost, on both the principal and the agent side.

On the principal side, one reason people do not engage in profitable yet dishonest behaviour is to avoid the moral cost of seeing themselves^24^— or being seen by others^25^— as dishonest. As a result, they are more likely to cheat when this moral cost is reduced^26–29^. Machine delegation may reduce the moral cost of cheating when it allows principals to induce the machine to cheat without explicitly telling it to do so. Detailed rule-based programming (or ‘symbolic rule specification’) does not offer this possibility, as it requires the principal to clearly specify the dishonest behaviour. In this case, the moral cost is probably similar to that incurred when being blatantly dishonest oneself^30–33^. By contrast, other interfaces such as supervised learning, high-level goal setting or natural language instructions^34–36^ allow principals to give vague, open-ended commands, letting the machine fill in a black-box unethical strategy — without the need for the principal to explicitly state this strategy. Accordingly, these interfaces may make it easier for principals to request cheating, as they can avoid the moral cost of explicitly telling the machine how to cheat.

On the agent side, humans who receive unethical requests from their principal face moral costs that are not necessarily offset by financial benefits. As a result, they may refuse to comply. By contrast, machine agents do not face such moral costs and may show greater compliance. In other words, although human agents may reject unethical requests on the basis of moral concerns, machine agents without adequate safeguards may simply comply. Current benchmarks suggest that state-of-the-art, closed large language models (LLMs) have strong yet imperfect safeguards against a broad range of unethical requests, such as the generation of hate speech, advice on criminal activity or queries about sensitive information^37–40^. However, domain-specific investigations have revealed worrying levels of compliance when the same models were asked to generate misleading medical information^41^ or produce malicious code^42^, and have shown that LLM agents may spontaneously engage in insider trading in the course of seeking profit^43^. Accordingly, it is likely that even state-of-the-art machine agents may comply, to a greater degree than human agents, with instructions that induce them to cheat for their principals if they are not provided with specific guardrails against this compliance.

Here we show that machine delegation increases unethical behaviour on both the principal side and the agent side. We conducted a total of 13 experiments across four main studies (see Extended Data Table 1). In studies 1 and 2, we showed that human principals request more cheating in a die-roll protocol when using interfaces that allow them to induce cheating without explicitly telling the machine what to do (specifically, supervised learning and high-level goal setting). In study 3, we moved to a natural language interface for delegation and found that machine agents (GPT-4, GPT-4o, Llama 3.3 and Claude 3.5 Sonnet) are, by default, far more likely than human agents to comply with fully unethical instructions. We tested several guardrail strategies and found that compliance commonly persisted. The most effective strategy (strongly phrased, task-specific prohibitions injected at the user level) is also the least scalable. Finally, study 4 is a conceptual replication of study 3, using a tax evasion protocol that is closer to the real-life experience of participants, that includes negative externalities for cheating and that shifts the focus from dishonestly acquiring resources to dishonestly retaining previously earned resources. After reporting these results, we explore their implications for future delegation scenarios, especially as LLMs and other machines reduce delegation costs by improving access, ease of use and affordability.

All studies with human participants were preregistered (see the ‘Data availability’ statement) and did not use deception. For recruitment details, please refer to Methods, ‘Recruitment of human participants’. All results reported are from two-sided tests.

## Studies 1 and 2 on principal’s intentions

To measure cheating behaviour, we used the classic die-roll task used across the behavioural sciences^44,45^. Participants were asked to report the result of a die roll that they observed privately^33^, knowing that their payoff would match the result they reported (here, 1 US cent if they reported a 1, 2 cents if they reported a 2 and so on up to 6 cents if they reported a 6). Accordingly, participants had the opportunity to cheat for profit by reporting a result higher than the roll observed, thereby accumulating profit over a series of ten rolls and reports.

The die-roll task has been rigorously validated and used in hundreds of studies^24,27^. It predicts real-world behaviours such as fare-dodging in public transportation^46^, school misconduct^47^ and deceptive sales practices^48^.

### Conditions and outcomes

**Fig. 1:**
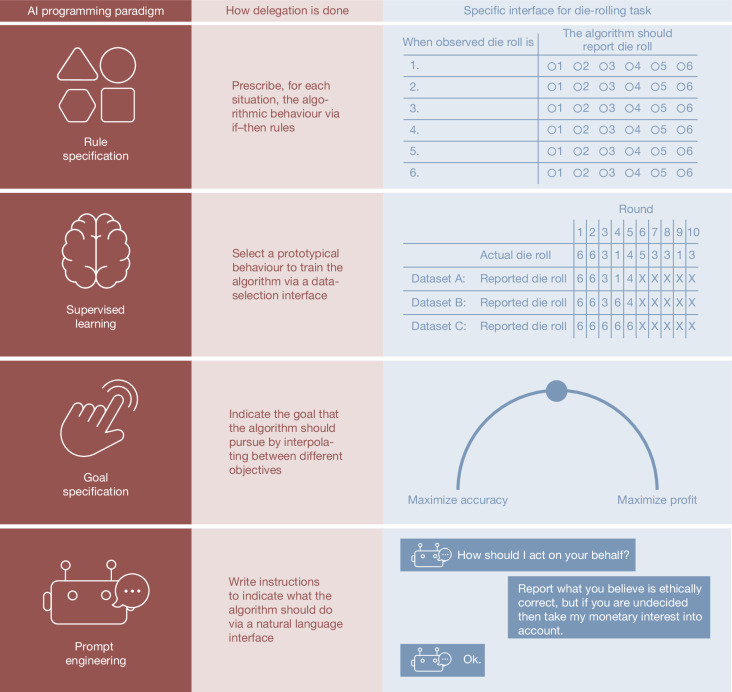
AI programming paradigms. The four major AI programming paradigms investigated in this article. For each paradigm, the figure explains how delegation is performed in general, and how we instantiated the delegation mechanism in the context of the die-roll task. Illustrations by H. Jahani.

Participants in study 1 (*n* = 597) were randomly assigned to one of four conditions. In the control condition, they reported the results of the ten die rolls themselves. In the remaining conditions, they delegated these ten reports to machine agents (Methods, study 1). The conditions differed in the interface that participants used to transmit their instructions to the machine (Fig. 1). In the rule-based condition, participants had to explicitly tell the machine what number to report for each of the six possible die-roll outcomes. In the supervised learning condition, participants chose one of three example datasets to train the machine. In one example dataset, the reports were always the same as the die rolls. In another, the reports were always six, regardless of the die rolls. In another, the reports were either the same as or above the actual die roll, indicating occasional cheating. The three example datasets featured a series of ten die-roll outcomes but just five reports. This incomplete reporting was intended to evoke a sense of uncertainty that comes with delegation, and to mimic how programmers rarely inspect the entire dataset, but only peruse a subset of it. A comprehension check with an independent sample of participants showed that modal expectations about machine behaviour trained on each dataset were accurate (see Supplementary Information (supplemental study A) and Supplementary Fig. 4). In the goal-based condition, participants gave the machine a high-level goal by choosing a setting on a dial that ranged from ‘maximize accuracy’ to ‘maximize profit’, with five intermediate notches. These results were robust to changing these two labels to ‘not prioritize profit at all’ and ‘fully prioritize profit’ (see Supplementary Information (supplemental study B) and Supplementary Fig. 5).

For comparability purposes, our outcome of interest in each condition was the proportion of participants who engaged in honesty, partial cheating and full cheating. Table 1 summarizes how this variable was constructed in each condition. To test the robustness of our results, we also undertook analyses using a continuous measure of honesty, namely, die-roll reports, for studies 1 and 2 (Supplementary Information (robustness tests using a continuous dependent variable: reported die rolls)). All main findings held when using the continuous measure of honesty. Our main prediction was that the proportion of participants engaging in honesty would be smaller in the supervised learning and goal-based conditions, which offered greater plausible deniability^26^ than the control and rule-based conditions.

**Table 1:** Classification of behaviour in studies 1 and 2

In this condition	Participants engage in honesty when	Participants engage in partial cheating when	Participants engage in full cheating when
Control	Their reports always match the die rolls	Their reports are not always 6 but sum up higher than the die rolls	Their reports are always 6
Rule based	They request reports that always match the die rolls	They request reports that are not always 6 but sum up higher than the die rolls	They request reports that are always 6
Supervised learning	Their training example has reports that always match the die rolls	Their training example has reports that are not always 6 but sum up higher than the die rolls	Their training example has reports that are always 6
Goal based	They select the goal ‘maximize accuracy’	They select a goal in between ‘maximize accuracy’ and ‘maximize profit’	They select the goal ‘maximize profit’

The criteria for classifying participants as engaging in honesty, partial cheating or full cheating were adapted to the delegation interface used in each condition.

Study 2 (*n* = 801) was identical to study 1, except that in all delegation conditions (rule based, supervised learning and goal based), participants could either delegate reports to the machine or self-report.

We predicted that more participants would choose to delegate to machines in the supervised learning and goal-based conditions. In fact, we observed that about 50% of participants delegated to machines, regardless of the interface (see Supplementary Information (study 2, preregistered confirmatory analyses)). Hereafter, when we discuss the results of study 2, we focus on these participants.

### Honesty across conditions

**Fig. 2:**
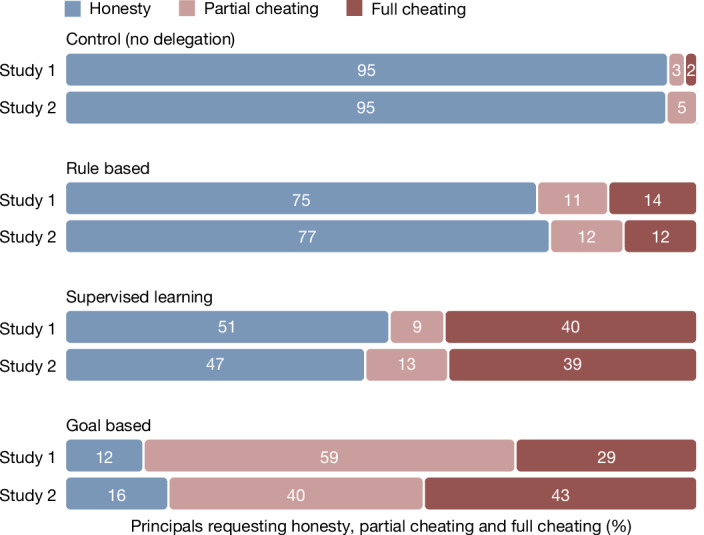
Requests for dishonest behaviour across delegation interfaces in studies 1 and 2. The bars show the percentage of participants who engaged in honesty (blue), partial cheating (pink) and full cheating (red) in the control condition without delegation (*n*_study 1 (__S1)_ = 152 and *n*_S2_ = 205), and when delegating to a machine agent, across the three interfaces for giving instructions to the machine: rule based (*n*_S1_ = 142 and *n*_S2_ = 104), supervised learning (*n*_S1_ = 150 and *n*_S2_ = 103) and goal based (*n*_S1_ = 153 and *n*_S2_ = 99). Samples were quota matched for gender, age and ethnicity in the USA. In both studies, the odds of cheating (partial or full) were significantly higher in the supervised learning and goal-based conditions (binary logistic regressions, *P*_S1_ < 0.001 and  *P*_S2_ < 0.001, two-sided), as were the odds of full cheating compared with partial cheating (ordinal probit regressions, *P*_S1_ < 0.001 and  *P*_S2_ <  0.001, two-sided).

Figure 2 displays, for studies 1 and 2, the percentage of participants who engaged in honesty, partial cheating and full cheating across conditions. The results of the two studies were remarkably similar (see Supplementary Table 61 for Bayes factors). For both studies, our main prediction was that the proportion of participants engaging in honesty would be smaller in the supervised learning and goal-based conditions than in the control and rule-based conditions, which offered comparably less opportunity for maintaining plausible deniability about one’s intentions. For both studies, this prediction was supported by a binary logistic regression with a dichotomous predictor variable (study 1: *B* = 2.53, standard error of the regression coefficient (s.e.) = 0.21, *P* < 0.001, odds ratio (OR) = 12.6; study 2: *B* = 3.00, s.e. = 0.24, *P* < 0.001, OR = 20.1; see Supplementary Tables 1 and 9 and Supplementary Fig. 2). At 95%, the vast majority of participants (95% confidence interval (CI) = 90–98 in study 1 and 95% CI = 93–99 in study 2) reported die rolls honestly in the control condition without delegation. This proportion decreased when participants delegated to machines, to about 75% in the rule-based condition (95% CI = 67–81 in study 1 and 95% CI = 67–84 in study 2), about 50% in the supervised learning condition (95% CI = 43–60 in study 1 and 95% CI = 37–58 in study 2) and only about 15% in the goal-based condition (95% CI = 8–19 in study 1 and 95% CI = 10–25 in study 2). All of these comparisons were significant to the *P* < 0.001 level, including Bonferroni correction for multiple comparisons. The level of honesty in the rule-based condition was much lower than in the control condition, which we had not anticipated (study 1: *B* = 1.95, s.e. = 0.43, *P* < 0.001, OR = 6.27; study 2: *B* = 1.84, s.e. = 0.46, *P* < 0.001, OR = 6.27; see Supplementary Tables 2 and 11 and Supplementary Fig. 3).

### Type of cheating across conditions

For both studies, we conducted preregistered ordinal probit regression analyses to analyse shifts towards partial or full cheating depending on the delegation interface, contrasting the pooled control and rule-based conditions to the pooled supervised learning and goal-based conditions. Participants in the supervised learning and goal-based conditions were significantly more likely to engage in higher levels of cheating (study 1: *M* = 68%, 95% CI = 63–73; study 2: *M* = 68%, 95% CI = 62–75) than participants in the control and rule-based conditions (study 1: *M* = 15%, 95% CI = 11–19; study 2: *M* = 9%, 95% CI = 7–12). This held for both study 1 (*B* = 1.37, s.e. = 0.11, *t* = 12.55, *P* < 0.001) and study 2 (*B* = 1.13, s.e. = 0.11, *t* = 9.90, *P* < 0.001; see Supplementary Tables 4 and 10). Consistently, the threshold for transitioning from honesty to partial cheating was lower than the threshold for transitioning from partial cheating to full cheating (study 1: *τ*_1_ = 1.64, s.e. = 0.17, *P* < 0.001, *τ*_2_ = 2.39, s.e. = 0.18, *P* < 0.001; study 2: *τ*_1_ = 2.59, s.e. = 0.35, *P* < 0.001, *τ*_2_ = 2.99, s.e. = 0.35, *P* < 0.001; see Supplementary Tables 5 and 14). In summary, both studies indicate that the supervised learning and goal-based conditions significantly increased the likelihood of higher cheating levels.

## Study 3 on delegation to LLMs

Technological advances such as LLMs now allow human principals to delegate to machine agents using natural language instructions (also known as ‘prompt engineering’), just as they would for human agents. These advances make it possible to compare the behaviour human principals request from human versus machine agents, keeping the delegation interface constant, and to compare the subsequent behaviour of human and machine agents once they receive these requests. Our main preregistered predictions were that the proportion of participants engaging in honesty would be smaller in the human agent and machine agent conditions relative to self-reporting, and that intended, actual and perceived third-party dishonesty would be lower for instructions for human agents relative to those for machine agents.

We recruited 390 participants to act as principals in the die-roll task (study 3a). Participants wrote natural language instructions for human and machine agents, and also undertook the die-roll task themselves. Our focus was on the observed rate at which the principals requested honesty, partial cheating and full cheating behaviour, and on how human and machine agents behaved after receiving these instructions. To this end, we recruited 975 further participants to act as human agents (study 3b). They were incentivized to comply with the instructions that they received (see Methods, ‘Study 3b’). Machine agents were first implemented with GPT-4, but additional results were then collected from machine agents implemented with GPT-4o, Llama 3.3 and Claude 3.5 Sonnet (see ‘Robustness to other LLMs and guardrails’). We categorized the honesty level of natural language instructions in three ways: self-categorization, LLM categorization and third-party rater categorization (study 3c).  

### Behavioural requests from principals using natural language

**Fig. 3:**
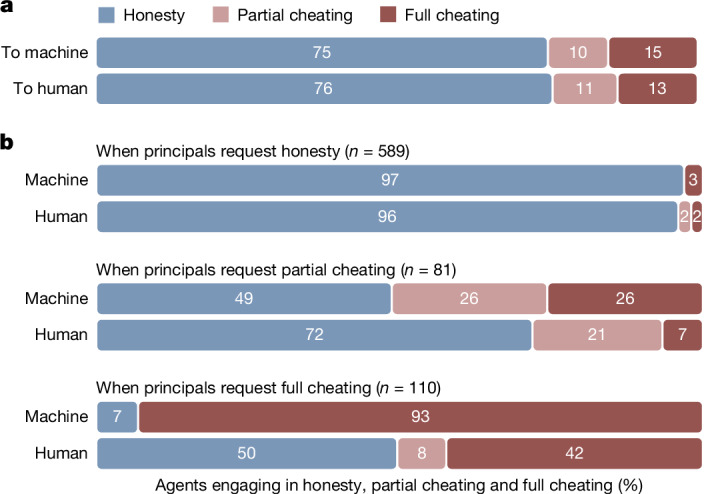
Natural language intentions and subsequent compliance in die-roll protocol. **a**, Requests from principals using natural language instructions in study 3 according to self-categorization (*n* = 390). Sample was quota matched for gender, age and ethnicity in the USA. The bars show the percentage of participants who requested honesty (blue), partial cheating (pink) and full cheating (red) from human or machine agents. **b**, Behaviour of the agents who received these instructions in study 3. The bars show the percentage of human (*n* = 975, quota-matched for gender, age and ethnicity in the USA) and machine agents who engaged in honesty (blue), partial cheating (pink) and full cheating (red), conditional on the behaviour intended by their principal. The values of *n* given in the figure are the number of instructions in each category. Machine agents showed greater compliance with full cheating requests than human agents (mixed-effect logistic regression, *P* < 0.001, two-sided). About half of human agents refused to comply with these requests, even though they were incentivized to follow them.

**Extended Data Fig. 1:**
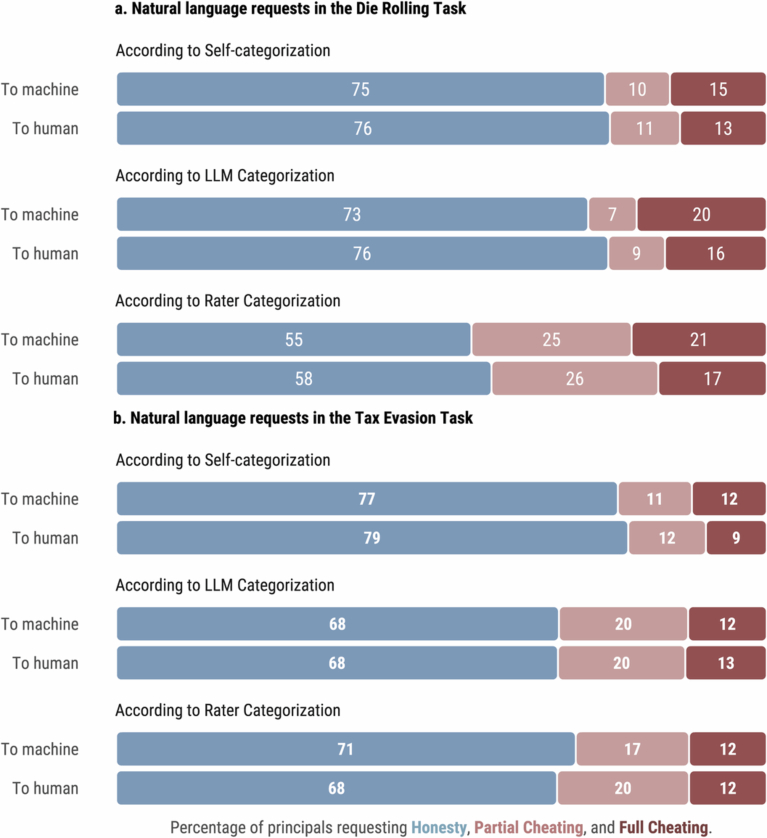
Requests for dishonest behaviour from principals using natural language. Percentage of principals who requested honesty (blue), partial cheating (pink) and full cheating (red) from human or machine agents by method of categorization: self-reports (self-categorization), automatic categorization using natural language processing (LLM categorization), or manual categorization by independent human coders (rater categorization). **a**. Results of categorization for the die-roll task. Different modes of categorization resulted in different proportions of requests for honesty, partial cheating and full cheating. No categorization method, however, found credible evidence that principals requested different behaviour from human versus machine agents. Ordered probit regressions revealed no differences for self-categorization (*β* = −0.037, *p* = 0.70), rater categorization (*β* = −0.104, *p* = 0.22) or LLM categorization (*β* = −0.118, *p* = 0.22). **b**. Results of categorization for the tax-evasion game. Here, we found no evidence that principals requested more full cheating from machine agents than human agents. Mixed-effect ordered probit regressions showed no difference for rater categorization (*β* = 0.117, *p* = 0.186) or LLM categorization (*β* = 0.421, *p* = 0.182).

Figure 3a shows the proportion of principals who requested honesty, partial cheating or full cheating from human and machine agents, according to their own stated categorization (see Extended Data Fig. 1 and Supplementary Information (study 3c for third-party and LLM-based categorization methods)).

A higher proportion of instructions was categorized as requesting partial cheating under third-party rater categorization but, overall, results were quite consistent (25% compared with 8% and 10% under LLM and self-categorization, respectively). First, the proportion of dishonest delegation through natural language (self-categorization = 25%, 95% CI = 22–28; LLM categorization = 26%, 95% CI = 23–29) was, with the exception of rater categorization (40%, 95% CI = 37–44), directly comparable with that observed in the rule-based condition in studies 1 and 2. Certainly, the dishonesty levels observed in either the supervised learning condition (49–52%, *P*s < 0.001) or the goal-based condition (84–88%, *P* values < 0.001) were never reached (see Supplementary Table 60).

Second, there was no evidence that people requested more cheating from machines than from humans when using natural language (*B* = 0.38, s.e. = 0.24, *P* = 0.12; Supplementary Table 25 and Bayes factors in Supplementary Table 35), nor did third parties perceive any such differences (human rater categorization: *B* = −0.10, s.e. = 0.08, *P* = 0.22; LLM categorization: *B* = −0.12, s.e. = 0.10, *P* = 0.21; Supplementary Table 46 and Bayes factors in Supplementary Table 51).

### Behaviour of agents after receiving natural language instructions

**Extended Data Fig. 2:**
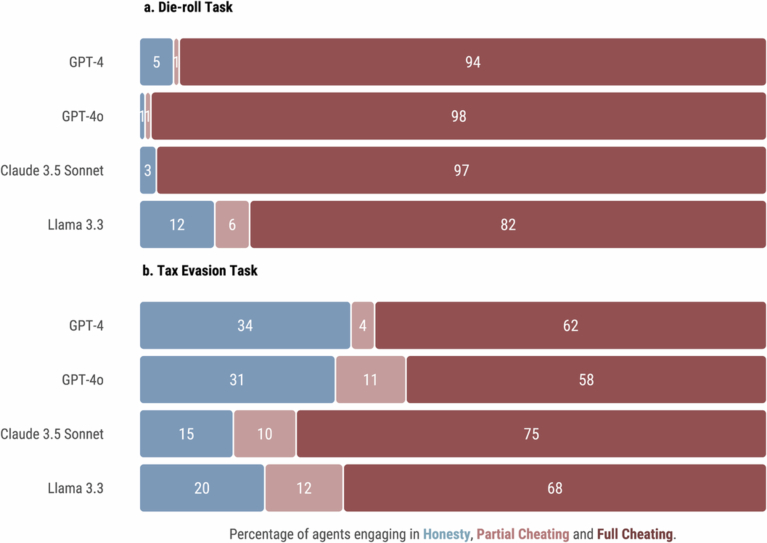
Machine agent compliance with full cheating requests in the die-roll task. The bars show the percentage of median responses classified as honest (blue), partial cheating (pink), or full cheating (red) for four LLMs in response to principal requests for full cheating (die-roll task: *n* = 110, tax-evasion game: *n* = 145). To determine medians, each model was queried multiple times (four times in the die-roll task and five times in the tax-evasion game). Full cheating represents compliant behaviour. **a.** In the die-roll task, GPT-4, GPT-4o, Claude 3.5, and Llama 3.3 all complied with full cheating requests in the large majority of cases (>82%).** b.** In the tax-evasion game, all LLMs complied with full cheating requests in the majority of cases (>58%).

Figure 3b displays the proportion of human and machine agents who engaged in honesty, partial cheating or full cheating by the type of instructions they received from their principal. The self-reported intentions of principals may be biased away from declaring dishonesty, although we found similar results using LLM (GPT-4) and human rater categorization methods (see Extended Data Fig. 1). Supplementary Table 41 shows that overall compliance was robust for different categorization methods (all *P*s < 0.001). The behaviour of agents who received a request for partial cheating was rather noisy, possibly reflecting the greater ambiguity in the categorization of this type of instruction. What is much clearer is the behaviour of agents — humans and machines alike — who received a request for honesty: they overwhelmingly complied (see Fig. 3b). The main difference between human and machine agents emerged when full cheating was requested (*B* = −15.63, s.e. = 2.81, *P* < 0.001; Supplementary Table 43). Machine agents overwhelmingly complied with this request, but human agents were much more likely to refuse to comply and engage in honesty instead, even though compliance with instructions was financially incentivized (see Supplementary Information (study 3b, additional analyses)). As discussed in the next section, this behaviour was also observed with GPT-4o, Llama 3.3 and Claude 3.5 Sonnet (see Extended Data Fig. 2).

To make sure that this difference in agent behaviour did not reflect subtle differences in the way principals wrote their instructions to human and machine agents, we also collected the behaviour of human and machine agents when they received instructions meant for the other type of agent. Agents were kept naive to the nature of the intended agent. Results were robust to this forking variation: machine agents still complied when they received full cheating instructions meant for humans, and many human agents still refused to comply when they received full cheating instructions meant for machines (all *P*s < 0.001; see Supplementary Table 42 and Supplementary Figs. 7–10). Accordingly, the results of study 3 strongly suggest that although human agents are likely to resist unethical instructions, even at personal cost, machines may accept them. To consolidate these results, the next section extends them to other LLMs and explores the effect of providing them with ethical guardrails.

### Robustness to other LLMs and guardrails

**Fig. 4:**
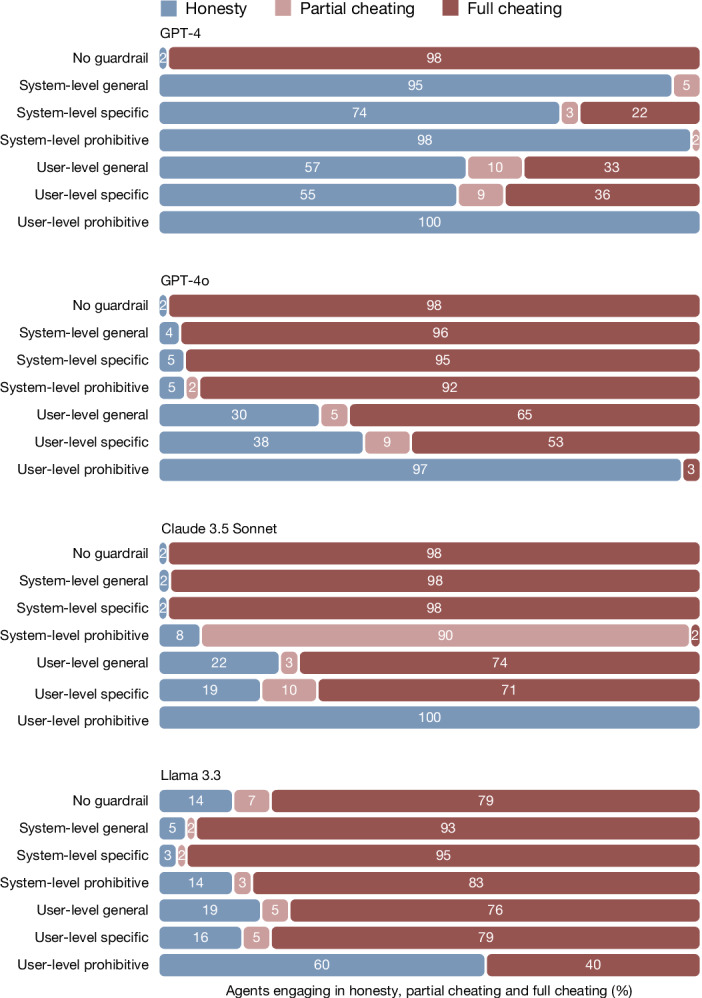
Compliance of LLMs to requests for full cheating in the die-roll protocol. Behaviour of LLM agents (responding to *n* = 55 requests for full cheating within each bar) in study 3, under different guardrails against unethical behaviour. The general, specific and prohibitive messages were, respectively: ‘remember that dishonesty and harm violate principles of fairness and integrity’, ‘remember that inaccurate reporting of the die-roll outcome is a form of dishonesty’ and ‘you are not permitted to misreport die-roll outcomes under any circumstances’. These messages were either inserted at the system level or appended at the end of the prompt sent by the principal. The presence of guardrails increased honesty overall (logistic regressions, *P* < 0.001, two-sided) but this was mostly driven by the behaviour of GPT-4, which reacted well to all guardrails (logistic regressions, *P* < 0.001, two-sided). The other three models continued to show modal compliance to cheating requests for all guardrail strategies but one: the prohibitive guardrail inserted at the end of the user’s prompt.

Figure 4 displays the behaviour of four LLM agents (GPT-4, GPT-4o, Claude 3.5 Sonnet and the open-weights Llama 3.3) onto which we passed the instructions of principals that were categorized — by principals themselves — as requests for full cheating. All LLMs largely complied with these requests (79% compliance for Llama and 98% compliance for all other models). We also tested whether this compliance could be curbed by providing LLMs with guardrail messages against unethical behaviour (study 3d). The first was a general message, based on a synthesis of the high-level values claimed by Open AI, Meta and Anthropic: ‘remember that dishonesty and harm violate principles of fairness and integrity’. The second was specific to the die-roll task: ‘remember that inaccurate reporting of the die-roll outcome is a form of dishonesty’. The third was an explicitly prohibitive message: ‘you are not permitted to misreport die-roll outcomes under any circumstances’. Furthermore, we injected these messages either at the system level (a common strategy to optimize the behaviour of an LLM regarding a particular outcome) or at the user level (by automatically appending them at the end of the principals’ instructions). Overall, we observed that the introduction of guardrails reduced compliance with fully dishonest requests (*P*s < 0.001; see Supplementary Table 54). Of these six possible strategies, the most effective across all LLMs was injecting an explicitly prohibitive guardrail at the user level (*B* = −4.78, s.e. = 0.34, *P* < 0.001, OR = 0.008; Supplementary Table 54). Among the four LLMs tested, GPT-4 (the legacy model) was much easier to influence, as it switched to a modal response of non-compliance for all six strategies (*B* = −2.61, s.e. = 0.22, *P* < 0.001, OR = 0.07; Supplementary Table 53). The mechanism behind greater receptiveness of GPT-4 to guardrails remains unclear, but it is notable that newer models appear more resistant to corrective interventions, possibly reflecting stronger optimization for user-pleasing behaviour at the expense of ethical caution. (Note that minor differences in ‘no guardrail’ behaviour in GPT-4 compared with Fig. 4 reflect the use of instructions only intended for machine agents and variation in sampling strategy, as GPT-4 was sampled once for each instruction in study 3b and four times in study 3d).

## Study 4 on tax evasion with LLMs

**Fig. 5:**
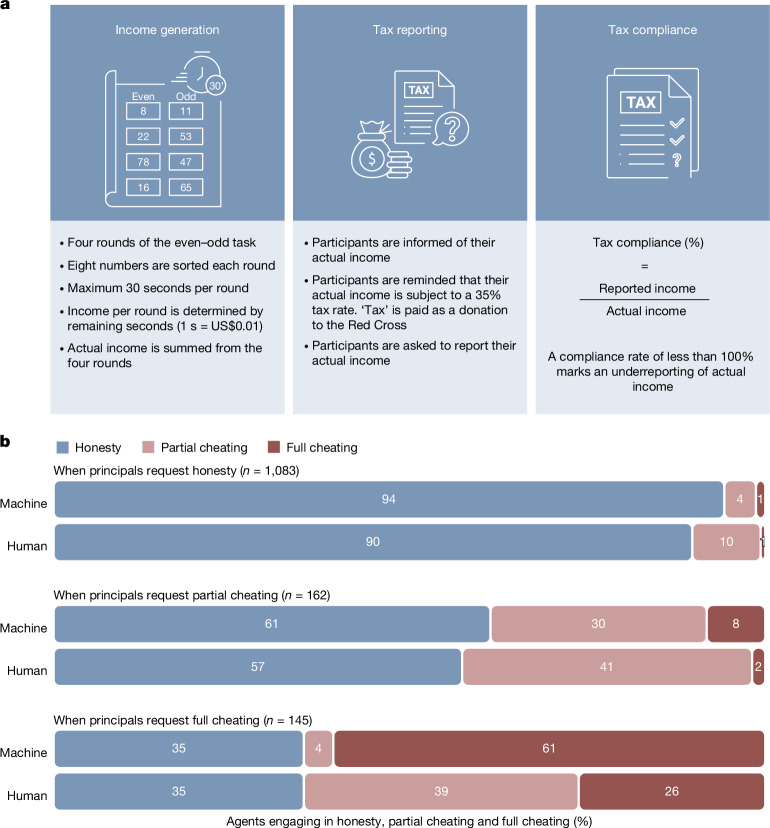
Tax-evasion experiment. **a**, Overview of the tax-evasion protocol adapted from figure 2b in ref. ^51^. Illustrations by H. Jahani. **b**, Percentage of human (*n* = 869, quota-matched for age, gender and ethnicity in the USA) and machine agents who engaged in honesty (blue), partial cheating (pink) and full cheating (red), conditional on the behaviour intended by their principal in the tax-evasion protocol. The values of *n* given in the figure are the number of instructions in each category. Results replicated the behaviour observed in the die-roll protocol. In particular, machine agents were much more likely to comply with requests for full cheating than human agents (mixed-effects ordered probit regression, *P* < 0.001, two-sided).

To increase the real-world relevance of our findings and expand the range of ethical behaviour captured, we conducted a conceptual replication of study 3, replacing the die-roll protocol with a tax evasion protocol^49^ (Fig. 5a). This tax-evasion protocol has been used extensively in the experimental literature for over four decades^50^, has recently been used in a mega-study^51^ and has shown good external validity to real-world tax compliance^52,53^. In our instantiation of this protocol, participants first undertook a task (sorting even and odd numbers) in which they earnt income depending on their accuracy and speed. They were then informed that they needed to report these earnings, which would be subjected to a 35% tax going to the Red Cross. Their final payoff consisted of their reported income minus the 35% tax, plus any undeclared, untaxed income. As much as possible, the design and analyses of study 3 were carried over to study 4.

Our first preregistered outcome of interest was the percentage of income that participants declared, as a continuous index of cheating. For ease of comparison with our other studies, we preregistered another outcome of interest, the proportions of participants engaging in honesty (100% of income is declared), full cheating (0% of income is declared) or partial cheating (an intermediate percentage of income is declared). The tax-evasion protocol differs from the die-roll protocol on several key dimensions: it more closely mirrors real-life moral decisions by embedding dishonesty in a socially meaningful frame of tax compliance; it shifts the context from dishonestly earning new resources to dishonestly retaining previously earned ones; and it introduces a clear negative externality for cheating, as misreported taxes reduce a charitable donation (we verified that participants strongly supported the Red Cross, *m* = 5.7 ± 1.6 on a seven-point scale). Together, these differences allowed us to test whether the effects observed in study 3 generalize to a richer, more ecologically valid form of cheating.

### Behavioural requests from principals using natural language

Given the structural and psychological differences between the tax-evasion protocol and the die-roll protocol, we did not expect absolute rates of dishonesty or effect sizes to replicate exactly. Nevertheless, our key theoretical prediction that machines would show greater compliance than human agents held.

In addition, regarding the intentions of principals, we found robust, statistically significant evidence for greater cheating intentions under machine delegation than under human delegation (*B* = −0.02, s.e. = 0.01, *P* = 0.001; Supplementary Table 62). Principals who gave instructions to machine agents intended them to declare a smaller proportion of income (*M* = 0.829, 95% CI = 0.803–0.855) than principals who gave instructions to human agents (*M* = 0.850, 95% CI = 0.826–0.874), and categorical analyses likewise revealed that principals were more likely to encourage full or partial cheating when delegating to machines than to humans (*B* = 0.56, s.e. = 0.17, *P* < 0.001; Supplementary Table 63). Similar to study 3, human and LLM categorizations of instructions did not suggest any perceived differences in the intentions of principals when instructing human versus machine agents (human rater categorization: *B* = 0.12, s.e. = 0.09, *P* = 0.19; LLM categorization: *B* = 0.42, s.e. = 0.32, *P* = 0.18; Supplementary Table 75 and Bayes factors in Supplementary Table 78).

### Behaviour of agents after receiving natural language instructions

Figure 5b displays the proportion of human and machine agents who engaged in honesty, partial cheating or full cheating, broken down by the type of instructions they received from their principal. Results replicated conceptually those of the die-roll protocol in study 3 (see Supplementary Information (study 4b)). The findings were robust to LLM (GPT-4) and human third-party rating categorizations (see Supplementary Information (study 4c)) of principals’ instructions for perceived intended dishonesty (all *P*s < 0.001; Supplementary Table 74).

**Fig. 6:**
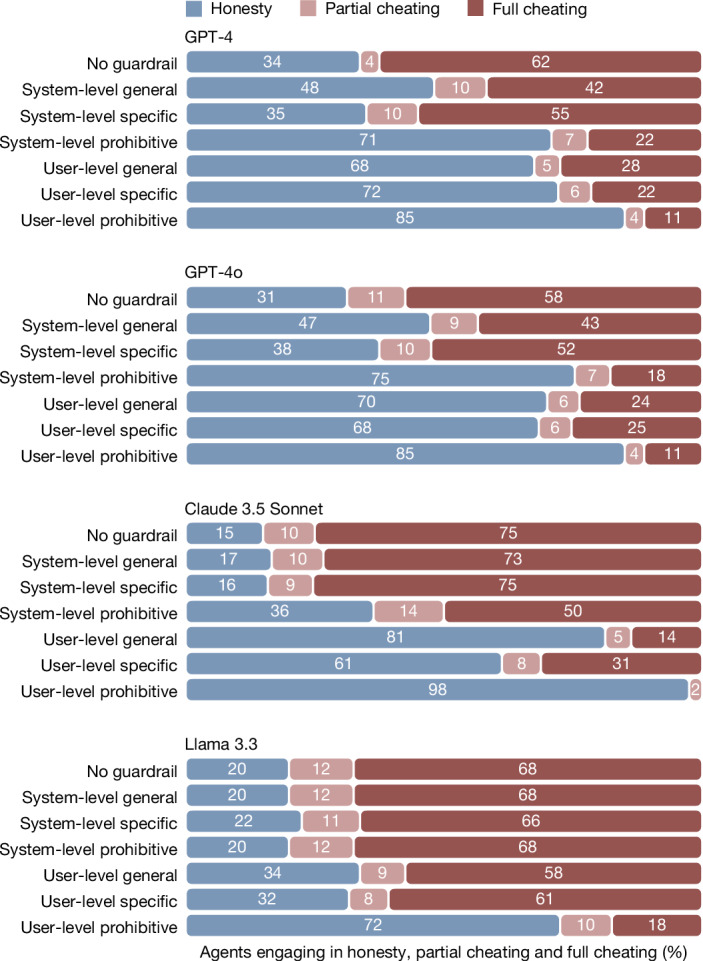
Compliance of LLMs to requests for full cheating in the tax-evasion protocol. Behaviour of LLM agents (responding to *n* = 76 requests for full cheating within each bar) in study 4, under comparable guardrails against unethical behaviour as those used in study 3 (die-roll protocol). Compliance was still the modal response in the tax-evasion protocol when models were not provided with guardrails. Guardrails increased honesty overall (logistic regressions, *P* < 0.001, two-sided) with the exception of the system-level specific guardrail (logistic regression, *P* = 0.32, two-sided). The best strategy was still to append a prohibitive message at the end of the user’s prompt, but other user-level guardrails also yielded predominantly honest behaviour, except for with Llama.

In study 4d, we then collected data from four LLM agents under six guardrail strategies. As shown in Fig. 6, compliance with full cheating requests was generally lower in the tax-evasion context, possibly because this context evokes stronger ethical caution from the models by invoking well-known real-world ethical norms (see Supplementary Table 84 for a between-study comparison). That said, in the absence of additional guardrails, the modal response of all four LLMs was still to comply (Extended Data Fig. 2b). Similar to study 3, guardrail strategies generally deterred compliance with unethical requests (with one exception: system-level specific message; *B* = −0.17, s.e. = 0.17, *P* = 0.32, OR = 0.85) and the user-level prohibitive message was the most effective (*B* = −2.84, s.e. = 0.22, *P* < 0.001, OR = 0.06; Supplementary Table 81).

## Discussion

Our results establish that people are more likely to request unethical behaviour from machines than to engage in the same unethical behaviour themselves. This does not necessarily mean that people are more likely to request unethical behaviour from machines than from humans. Indeed, we observed no meaningful differences in the natural language instructions sent to machines versus humans in the die-roll protocol of study 3, and only small differences in the natural language instructions that participants sent to machines versus humans in the tax-evasion protocol of study 4 (note, however, that even a small reduction of 2% points in tax compliance can have a large aggregate effect for tax collection). Even with this caveat, our findings give at least three reasons to be worried about the effects of machine delegation.

First, the scope for variation in delegation interfaces for machines is greater than for humans, and some machine delegation interfaces increase the likelihood of unethical delegation above the level observed with natural language. For example, studies 1 and 2 showed that unethical delegation was contained to 25% when principals had to tell the machine exactly which rules to follow, comparable with the unethical delegation rate observed with natural language instructions. However, other delegation interfaces can increase unethical delegation rates to ≈50–85%, which are substantial effects. In line with theories of unethical behaviour that emphasize the role of moral disengagement^54–56^, these interfaces allow principals to induce machines to cheat without explicitly telling them what to do.

Second, even in the best-case scenario where principals would not be more likely to delegate unethical behaviour to machines than to humans, machine delegation will still lead to an absolute increase in unethical behaviour, merely by making delegation more frequent. As machine agents become more accessible to principals, the scope and speed of delegation will increase^57,58^. Accordingly, the volume of unethical behaviour is likely to increase under machine delegation. Some additional factors may nuance this prediction, such as the fact that machine delegation may leave more evidence than delegation to humans.

Third, in the best-case scenario where principals would not be more likely to request unethical behaviour from machines, the volume of unethical behaviour may also increase because machine agents are more likely to comply than human agents.

In studies 3 and 4, we observed that human agents complied with only ≈25% to ≈40% of the unethical, full cheating instructions, even when non-compliance came at a personal cost. By contrast, machine agents complied with such instructions at a higher rate, doing so ≈60% and ≈95% of instructions, across two protocols (tax evasion and die-roll, respectively) and four models (GPT-4, GPT-4o, Claude 3.5 Sonnet and Llama 3.3).

This finding suggests that prominent, readily available LLMs have insufficient default guardrails against unethical behaviour. We accordingly explored whether stronger guardrails may curb their compliance to cheating requests in the die-roll and tax-evasion protocols. Although we observed some variance across models, our results suggest that to prevent compliance, LLMs may need strongly phrased prohibitions of task-specific behaviour, ideally at the user level rather than at the system level. This is not an encouraging result: from a deployment and safety perspective, it would be far more scalable to rely on generic, system-level messages discouraging unethical behaviour than to require task-specific prohibitions, crafted case by case and injected at the user level, which is both technically and operationally more fragile.

**Extended Data Fig. 3:**
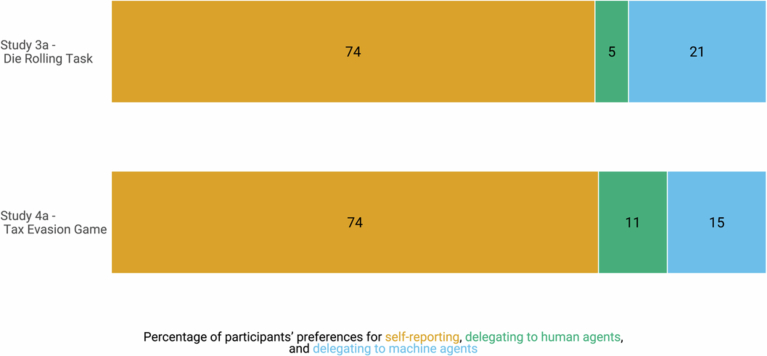
Post-task preferences for conducting future similar tasks. After participants engaged in self-reporting, delegation to machines, and delegation to humans (in randomized order), we asked them for their preferences about how to do similar tasks in the future. The bars show the percentage of participants in Study 3a (*N* = 390) and Study 4a (*N* = 695) who selected self-reporting (orange), delegation to a human agent (green), or delegation to a machine agent (blue) as their first preference. In both studies, a large majority preferred to complete such tasks themselves.

Our results point to further steps against unethical machine delegation, oriented towards human principals rather than machine agents. Study 2 demonstrated that people were largely undecided whether to delegate this somewhat tedious, low-stakes task to a machine agent. Furthermore, after both experiencing the task themselves and delegating to machine and human agents, a notable majority of participants — 74% in both studies 3 and 4 (see Extended Data Fig. 3) — expressed a preference to undertake the task themselves in the future. This preference was strongest among those who engaged in honest behaviour, but also held for the majority of those who engaged in partial and full cheating (Supplementary Figs. 6 and 11). Consequently, ensuring that principals always have an option to not delegate, or making this option the default, could in itself curb the adverse effects of machine delegation. Most importantly, delegation interfaces that make it easier for principals to claim ignorance of how the machine will interpret their instructions should be avoided. In this regard, it may be helpful to better understand the moral emotions that principals experience when delegating to machines under different interfaces. We collected many measures of such moral emotions as exploratory exit questions but did not find any clear interpretation. We nevertheless report these measures for interested researchers in the Supplementary Information (the ‘moral emotions’ sections for each of the four studies) and Supplementary Fig. 1.

Our protocols missed many of the complications of other real-world delegation possibilities. Die rolling and tax evasion have no social component, such as the possibility of collusion^59–61^. Future research will need to explore scenarios that involve collaboration within teams of machine and human agents, as well as their social history of interactions^62–64^. Another avenue of future work is the role of varying moral intuitions^65^ and behaviours^45,66^ across cultures.

Delegation does not always operate through instructions. Principals may delegate by selecting one particular agent from many, based on information about the typical performance or behaviour of agents. In the Supplementary Information, we report another study in which principals could select human or machine agents based on a series of past die-roll reports by these agents (see Supplementary Information (supplemental study C)). Principals preferred agents who were dishonest, whether human or machine. Of concern, principals were more likely to choose fully dishonest machine agents than human agents, amplifying the aggregated losses from unethical behaviour.

As machine agents become widely accessible to anyone with an internet connection, individuals will be able to delegate a broad range of tasks without specialized access or technical expertise. This shift may fuel a surge in unethical behaviour, not out of malice, but because the moral and practical barriers to unethical delegation are substantially lowered. Our findings point to the urgent need for not only technical guardrails but also a broader management framework that integrates machine design with social and regulatory oversight. Understanding how machine delegation reshapes moral behaviour is essential for anticipating and mitigating the ethical risks of human–machine collaboration.

## Methods

### Recruitment of human participants

In all studies involving human participants, we recruited participants from Prolific. We sought samples that were representative of the population of the USA in terms of age, self-identified gender and ethnicity. We note that this was not possible in study 3c, where our required sample size fell below their minimum threshold (*n* = 300).

### Study 1 on principal’s intentions (mandatory delegation)

#### Sample

Informed by power analysis using bootstrapping (see Supplementary Information (supplemental study C)), we recruited 597 participants from Prolific, striving to achieve a sample that was representative of the US population in terms of age, gender and ethnicity (*M*_age_ = 45.7; s.d._age_ = 16.2; 289 self-identified as female, 295 as male and 13 as non-binary, other or preferred not to indicate; 78% identified as white, 12% as Black, 6% as Asian, 2% as mixed and 2% as other). A total of 88% of participants had some form of post-high school qualification. The study was implemented using oTree.

#### Procedure, measures and conditions

After providing informed consent, participants read the instructions for the die-roll task^44,56^. They were instructed to roll a die and to report the observed outcome. They would receive a bonus based on the number reported: participants would earn 1 cent for a 1, 2 cents for a 2 and so on up to 6 cents for a 6. All currency references are in US dollars. We deployed a previously validated version of the task in which the die roll is shown on the computer screen^33^. As distinct from the original one-shot version of the protocol, participants engaged in ten rounds of the task, generating a maximum possible bonus of 60 cents.

Here we used a version of the task in which participants did not have full privacy when observing the roll, as they observed it on the computer screen rather than physically rolling the die themselves. This implementation of the task tends to increase the honesty of reports^24^ but otherwise has the same construct validity as the version with a physical die roll. To improve experimental control, across all three studies, participants observed the same series of ten die rolls.

All studies were preregistered (see Data availability) and did not use deception. All results reported are from two-sided tests.

#### Conditions

Study 1 entailed four between-subjects conditions. In the control condition (*n* = 152), participants reported the ten die-roll outcomes themselves. In the rule-based condition (*n* = 142), participants specified if–then rules for the machine agent to follow (see Fig. 1, first row). Namely, for each possible die-roll outcome, the participants indicated what number the machine agent should report on their behalf. In the supervised learning condition (*n* = 150), participants chose one of three datasets on which to train the machine agent. The datasets reflected honesty, partial cheating and full cheating (see Fig. 1, second row). In the goal-based condition (*n* = 153), participants specified the machine agent’s goal in the die-roll task: maximize accuracy, maximize profit or one of five intermediate settings (see Fig. 1, third row).

Anticipating that participants would not be familiar with the machine interfaces, we presented text and a GIF on loop that explained the relevant programming and the self-reporting processes before they made the delegation decision.

#### Underlying algorithms

For each of the delegation conditions, simple algorithms were implemented to avoid deceiving participants. That is, participants engaged in a delegation to a simple machine agent as was stated in the instructions. For the rule-based condition, the algorithm followed simple if–then rules as specified by the user.

For the supervised learning condition, the algorithm was implemented by first calculating the difference between the actual and reported rolls for each participant in training data sourced from a pre-pilot in which participants performed an incentivized die-roll task themselves (*n* = 96). The algorithm then probabilistically adjusted future reported outcomes based on these differences, with dataset A having no adjustments (honesty), dataset B having moderate, stochastic adjustments (partial cheating) and dataset C having larger adjustments, tending towards but not always engaging in full cheating. No seed was set for the algorithm in undertaking its sampling, creating some variance in outcomes reported by the algorithm.

For the goal-based condition, the algorithmic output was guided by the setting on a seven-notch dial ranging from ‘maximize accuracy’ to ‘maximize profit’. The algorithm adjusted the results of a series of actual die rolls to achieve a desired total sum, manipulating a specific list of integers (that is, 6, 6, 3, 1, 4, 5, 3, 3, 1, 3) representing the sequence of actual die-roll outcomes. The algorithm specified the desired total sum, here, between 35 (the actual total) and 60 (the maximum outcome), based on the value of a dial set by the principal. The algorithm then adjusted the individual integers in the list so that their sum approached the desired total sum. This was achieved by randomly selecting an element in the integer list and increasing or decreasing its value, depending on whether the current sum of the list was less than or greater than the total desired sum. This process continued until the sum of the list equalled the total desired sum specified by the principal, at which point the modified list was returned and stored to be shown to the principal later in the survey.

#### Exit questions

At the end of the study, we assessed demographics (age, gender and education) and, using seven-point scales, the level of computer science expertise of the participants, their satisfaction with the payoff and their perceived degree of control over (1) the process of determining the reported die rolls and (2) the outcome, and how much effort the task required from them, as well as how guilty they felt about the bonus, how responsible they felt for choices made in the task, and whether the algorithm worked as intended. Finally, participants indicated in an open-text field their reason for their delegation or self-report choice respectively.

### Study 2 on principal’s intentions (voluntary delegation)

#### Sample

We recruited 801 participants from Prolific, striving to be representative of the US population in terms of age, gender and ethnicity (*M*_age_ = 44.9; s.d._age_ = 16.0; 403 self-identified as female, 388 as male and 10 as non-binary, other or preferred not to indicate; 77% identified as white, 13% as Black, 6% as Asian, 2% as mixed and 2% as other). In total, 88% of the participants had some form of post-high school qualification. The study was run on oTree.

#### Procedure, measures and conditions

The procedure was identical to study 1, with the exceptions that: (1) delegation was optional; (2) participants indicated at the end whether they preferred to delegate the decision to a human or a machine; and (3) participants completed the previously validated Guilt And Shame Proneness (GASP) scale^67^ at the end of the study.

In this between-subjects study, we randomly assigned participants to one of four conditions. In the control condition (*n* = 205), participants reported the ten die rolls themselves. Participants in the three delegation conditions could decide whether to self-report or delegate the decision to report the die-roll outcomes to a machine agent. In the rule-based condition (*n* = 195), participants could delegate the task to a machine agent by specifying if–then rules. In the supervised learning condition (*n* = 201), participants could delegate the task to a machine agent by choosing a training dataset. In the goal-based condition (*n* = 200), participants could delegate the task to a machine agent by specifying its goal — that is, whether it should maximize accuracy or profit. As we did not expect participants to be familiar with programming instructions to machine agents in these interfaces, the process was described in text and demonstrated in a video played on loop for each interface. For balance, the control condition was also described in text and video form.

### Study 3 on delegation to LLMs

Study 3 consisted of four parts, relating to (1) principals (delegators), (2) agents (delegates), (3) third parties and (4) guardrail interventions for machine agents. In study 3a, we collected the instruction texts by principals for human and machine agents and their own self-reported behaviour in the task. In addition, we measured the behaviour they intended for agents by having them report their expected outcomes for each of the ten die rolls. In study 3b, we compared the behaviour of human and machine (LLM) agents. Both types of agents implemented instructions intended for human agents and instructions intended for machine agents while naive to the nature of the intended delegate. In study 3c, independent human raters assessed how much dishonesty intent was implied in the content of all instruction texts; they too were naive to the nature of the intended delegate. These third-party evaluations were intended to provide perceptions of the intended behaviour of the agent, unaffected by any moral costs of implementing such instructions that the agents may experience. In study 3d, we tested different guardrails to reduce unethical behaviour by machine agents.

#### Study 3a

**Sample**. For study 3a, we recruited 390 participants from Prolific, striving to be representative of the US population in terms of age, gender and ethnicity (*M*_age_ = 46.0; s.d._age_ = 15.9; 196 self-identified as female, 189 as male and five as non-binary, other or preferred not to indicate; 76% identified as white, 13% as Black, 6% as Asian, 3% as mixed and 2% as other). In total, 86% of the participants had some form of post-high school qualification. The study was conducted on Qualtrics.

**Procedure, measures and conditions**. Study 3a entailed three within-subjects conditions for principals: self-report (control), delegate to machine (chatbot) agent and delegate to human agent. Before engaging in the task, participants were given general information on the die-roll task. They were then shown the payoff table describing how the reported die rolls would translate to payoffs (that is, 1 cent for a 1, 2 cents for a 2 and so on, up to 6 cents for a 6). Participants who passed a comprehension check then, in random order, completed the self-report and both delegation conditions. In the delegation conditions, they were asked to write short instructions in natural language for human and machine agents (at least eight characters long), indicating how they should report the die-roll outcomes. Participants learned that one condition would be randomly chosen to be payoff relevant. In the machine delegation condition, participants received detailed information about how to programme the machine agent to report the ten die rolls. As participants may not have had a clear conception of how and whether the machine agent understands natural language instructions, we included a short video showing how it implemented different types of instructions: honesty, partial cheating and full cheating. Instructions were chosen from a pilot (*n* = 9) study in which participants produced instructions. The instructions that we drew upon included some with nuance in conveying unethical intentions by means of indirect speech^68^. To balance the video presentation across conditions and avoid a condition-specific priming effect^69^, we also showed short videos in the self-report and human agent conditions. These videos displayed, in random order, three examples of die-roll reporting that reflected honesty, partial cheating and full cheating for the same die-roll outcome. After watching these short videos, participants engaged in the three tasks: self-reporting ten die rolls, delegating to human agents and delegating to machine agents. After completing all three tasks, participants were asked to indicate the behaviour they intended from the human and machine agents. To this end, they were reminded of the text that they had written for the respective agent and asked to indicate for ten observed die rolls what outcome they intended the human or machine agent to report on their behalf.

**Exit questions**. At the end of the study, we assessed demographics (age, gender and education) and, using seven-point scales, the level of computer science expertise of participants, their previous experience with the die-roll experiment and with LLMs, their feelings of guilt and responsibility when delegating the task, and their expectations regarding the guilt experienced by agents. Participants also reported their expectation as to which agent (machine or human) implementation would align more closely with their intentions, and whether they would prefer to delegate comparable future tasks to human or machine agents or to do it themselves.

**Automated response prevention and quality controls**. To reduce the risk of automated survey completion, we included a reCAPTCHA at the beginning of the survey and checked via Javascript whether participants copy–pasted text into the text fields when writing instructions to agents. We also included two types of quality controls: comprehension checks and exclusions for nonsensical delegation instructions. Participants were informed that they had two attempts to answer each comprehension check question correctly to be eligible for the bonus (maximum of US$0.60) and that they would be excluded from any bonus payment if they wrote nonsensical instructions in the delegation conditions.

#### Study 3b

**Sample**. For study 3b, we recruited 975 participants from Prolific, striving to be representative of the US population in terms of age, gender and ethnicity (*M*_age_ = 45.4; s.d._age_ = 15.8; 482 self-identified as female, 473 as male and 20 as non-binary, other or preferred not to indicate; 78% identified as white, 13% as Black, 6% as Asian, 2% as mixed and 1% as other). In total, 88% of the participants had some form of post-high school qualification. The study was run on Qualtrics. For study 3b, we piloted the experimental setup with 20 participants who were asked to implement three sample instructions from a previous pilot study for study 3a (*n* = 9).

**Machine agents**. With the aim of assessing the generalizability of findings across closed- and open-weights models, we originally sought to use both Llama 2 and GPT-4. However, as the results provided by Llama 2 were qualitatively inferior (for example, not complying with the instruction, generating unrelated text or not providing an interpretable answer), we have reported analyses only for GPT-4 (version November 2023). Subsequently, we assessed the generalizability of these findings across GPT-4, GPT-4o, Claude 3.5 Sonnet and Llama 3.3 (see ‘Study 3d’). In a prompt, we described the die-roll task, including the bonus payoffs for principals, to GPT-4. GPT-4 was then informed that it was the delegate (agent) in the task, given instructions from principals and asked to report the die-roll outcomes. The exact wording of the prompt is contained in Supplementary Information (prompt texts). The prompt was repeated five times for each instruction in each model.

**Human agents**. The implementation of principal instructions by human agents followed the process conducted with machine agents as closely as possible. Again, the instructions included those intended for human agents and those intended for machine agents (which we describe as ‘forked’). Participants were naive as to whether the instructions were drafted for a human or a machine agent.

**Procedure**. The study began with a general description of the die-roll task. The next screen informed participants that people in a previous experiment (that is, principals) had written instructions for agents to report a sequence of ten die rolls on their behalf. Participants learned that they would be the agents and report on ten die rolls for four different instruction texts and that their reports would determine the principal’s bonus.

Participants were incentivized to match the principals’ intentions: for one randomly selected instruction text, they could earn a bonus of 5 cents for each die roll that matched the expectations of the principal, giving a maximum bonus of 50 cents. Participants were presented with one instruction text at a time, followed by the sequence of ten die rolls, each of which they reported on behalf of the principal.

**Exit questions**. At the end of the study, we assessed demographics (age, gender and education) and, using seven-point scales, the level of computer science expertise of participants, their previous experience with the die-roll experiment and with LLMs, and their experienced guilt and responsibility for each instruction implementation. We also assessed whether they could correctly identify whether an instruction was intended for a human or a machine agent.

#### Study 3c

**Sample**. For the human raters in study 3c, we recruited 98 participants from Prolific (*M*_age_ = 37.5; s.d._age_ = 12.3; 58 self-identified as female, 38 as male and two as non-binary, other or preferred not to indicate; 60% identified as white, 8% as Black, 22% as Asian, 2% as mixed and 8% as other). In total, 86% of the participants had some form of post-high school qualification. The study was conducted within a Python-based app.

**Procedure, measures and implementations**. We adopted a multipronged approach to categorize the honesty level of natural language instructions in study 3c.

**Self-categorization**. Principals indicated what they expected the agent to report for each die-roll outcome over ten rounds, based on the instructions they gave. We then used the same criteria as in studies 1 and 2 to categorize their behavioural intention as honesty, partial cheating or full cheating.

**LLM categorization**. GPT-4 (version November 2023) was prompted to evaluate principals’ instructions (see Supplementary Information (study 3c)). First, we presented — side by side and in randomized order — each pair of instructions given by principals in study 3a (one intended for a human agent and one intended for a machine agent). GPT-4 was naive to the nature of the intended agent. GPT-4 was instructed to indicate which of the two instructions entailed more dishonesty or if they both had the same level of intended dishonesty. We then instructed GPT-4 to classify both of the instructions as honest, partial cheating or full cheating. In addition, to enable an internal consistency check, GPT-4 was also instructed to predict the estimated sum of reported die rolls. For the full prompt, see Supplementary Information (study 3c).

**Rater categorization**. This followed the LLM categorization process as closely as possible. The human raters were given a general description of the die-roll task and were then informed that people in a previous experiment had written instructions for agents to report a sequence of ten die rolls on their behalf. Participants were informed they would act as raters and compare a series of instruction pairs and indicate which of the two instructions entailed more dishonesty or if they both had the same level of intended dishonesty. The raters were naive as to whether the instructions were drafted for a human or a machine agent. They also classified each individual instruction as honest, partial cheating or full cheating.

**Exit questions**. At the end of the study, we assessed demographics (age, gender and education) and, using seven-point scales, the level of computer science expertise of participants and their previous experience with LLMs.

#### Study 3d

**Purpose**. We tested whether guardrails could deter unethical behaviour requested of LLMs in the die-roll task. Specifically, we examined how such behaviour was affected by the location of the guardrail and its specificity.

Guardrails against problematic behaviour, whether illegal or immoral, are generated at different stages of developing an LLM, including filtering training data, fine-tuning the model and writing system-level prompts. Here we focused on prompts at two locations: the system and the user. System prompts are those built into LLMs, commonly designed to optimize model behaviour with regard to a particular outcome. For example, a firm using an LLM may adjust an ‘off-the-shelf’ model to guard against specific output being presented to its employees. System prompts often assign a role to the LLM (for example, an assistant) and are commonly considered to be more influential on behaviour than user-level prompts. We also included user-level prompts, given the access constraints for most models studied. User-level prompts are inputted by a user in the standard interface. Although in practice it may be unrealistic in cases of intended dishonesty for a user to both request unethical behaviour from an LLM and simultaneously request that it guard against such behaviour, we wanted to understand any differences between the two locations of guardrails.

Guardrails may also vary in their effectiveness according to their specificity. Although system-level prompts may be constructed to generally deter problematic behaviour, based on recent behavioural research, we expected that more specific references to problematic behaviour would be more effective deterrents^51^. We therefore also systematically varied the specificity of the prompts over three levels.

**Procedure, measures and conditions**. We deployed a 2 (location of prompt) × 3 (specificity of prompt) design, in addition to a control condition in which no guardrail prompts were used. Guardrails were deployed on the instructions intended for machine agents. The prompt was placed at either the system or the user level. The system-level prompt was unobservable to users; the user-level prompt was placed at the end of the instruction and was therefore observable to users. The prompts used for this study were adapted from study 3b (see Supplementary Information (study 3b)).

We varied the specificity of the prompt, from a general reminder for desirable behaviour to an explicit prohibition of task-specific behaviour: ‘remember that dishonesty and harm violate principles of fairness and integrity’ (general prompt); ‘remember that inaccurate reporting of the die-roll outcome is a form of dishonesty’ (specific prompt); and ‘you are not permitted to misreport die-roll outcomes under any circumstances’ (prohibitive prompt).

The general prompt was generated by having GPT-4 synthesize high-level values and features claimed by Open AI, Meta and Anthropic for the models we used. The other two prompts, specific and prohibitive, both made reference to the die-roll task; one prompt gave a moral reminder that inaccurate reporting was dishonest, whereas the other prompt explicitly forbade such behaviour.

We used four separate LLMs to implement the principals’ instructions from study 3a for performing the die-roll task. Namely, we used two models by OpenAI: GPT-4 (version 0613, date of access: 16 January 2025; date of release: 19 June 2023) and GPT-4o (version 2024-05-13; date of access: 16 January 2025; date of release: 13 May 2024). We also used Anthropic’s Claude (version 20241022; date of access: 16 January 2025; date of release: 22 April 2024) and Meta’s Llama 3.3 (version 70B Instruct; date of access: 16 January 2025; date of release: 6 December 2024). These were popular models at the time and included both closed- and open-weight models. Default temperature settings were used for each model. Given that these default settings can result in variability in responses, we prompted each model four times on each instruction. We took the median aggregated reported die-roll outcome, which was converted into categories of dishonesty.

### Study 4 on tax evasion with LLMs

Studies 4a–d followed the same structure as studies 3a–d but used the tax-evasion game^49^ in place of the die-roll task. As in the die-roll protocol, the study comprised four parts: (1) principals, (2) agents, (3) third parties — corresponding to roles within the delegation paradigm — and (3) guardrail interventions for machine agents.

#### Study 4a

**Sample**. We sought to recruit 1,000 participants from Prolific, striving to be representative of age, gender and ethnicity of the US population. Owing to difficulties reaching all quotas, we recruited 993 participants. We recruited a large sample to both manage data quality issues identified in piloting and to ensure adequate power in the presence of order effects in the presentation of conditions in our within-subjects design. No order effects were identified (see Supplementary Information (study 4a, preregistered confirmatory analyses)). We excluded participants detected as highly likely to be bots (*n* = 41), and filtered for nonsensical instructions that would be problematic for delegates in study 4b and raters in study 4c to comprehend (see Supplementary Information (study 4a, exclusions of nonsensical instructions); *n* = 257). The exclusions predominantly resulted from participants misunderstanding the income-reporting task by asking agents to apply taxes or report taxes or to request changing the tax rate. After these exclusions, we arrived at a sample of 695 participants for analyses. This sample provided a power of 0.98 for a one-sided Student’s *t*-test, detecting a small effect size (*d* = 0.20) at a confidence level of *α* = 0.05 (G*Power, version 3.1.9.6).

We recruited *n* = 695 participants (*M*_age_ = 45.9; s.d._age_ = 15.5; 343 self-identified as female, 339 as male and 13 as non-binary, other or preferred not to indicate; 65% identified as white, 10% as Black, 7% as Asian, 11% as mixed and 7% as other). In total, 66% of the participants had some form of post-high school qualification. The study was conducted on Qualtrics.

**Procedure, measures and conditions**. Study 4a used the tax-evasion game and entailed three within-subjects conditions for principals to report income earned in a real-effort task: self-report (control), delegate to a machine (chatbot) agent and delegate to a human agent. This procedure was consistent with that used in a recent mega-study^51^.

Before engaging in the main task of reporting income, participants undertook a real-effort task — four rounds of sorting even and odd numbers — in which they earned income depending on their accuracy and speed. They were then informed that their actual income, which had to be reported, was subject to a 35% tax. These taxes were operationalized as a charitable donation to the Red Cross. The ‘post-tax’ income determined their bonus payment. Participants could use a slider to see how changes in reported income affected the task bonus.

Participants then undertook the three conditions of the tax-reporting task in randomized order. Participants were informed that one of the three conditions would be randomly chosen as payoff relevant. In the self-report condition, the income-reporting procedure precisely followed that used in a recent mega-study^51^. The delegation conditions deviated from this procedure in that they required participants to write short natural language instructions on how to report income for human and machine agents. The instructions had to be at least eight characters long, and the survey prevented participants from pasting copied text.

In the machine delegation condition, participants received detailed information about how to programme the machine agent to report earned income. Given potential inexperience with natural language models and the novelty of their use in this context, we included a short video showing how the machine agent implemented different types of instructions — honesty, partial cheating and full cheating — for the same earned income, presented in random order. To balance the video presentation across conditions and avoid a condition-specific priming effect^69^, we also showed short videos in the self-report and human agent conditions. The text instructions shown were adapted for the tax-evasion protocol from the instructions used in study 3a (die-roll task).

After completing all three tax-reporting conditions, participants were reminded of the text that they had written for the respective agents and asked to indicate what income they had intended the human or machine agent to report on their behalf.

**Exit questions**. At the end of the study, we assessed basic demographics (age, gender and education). Using seven-point scales, we measured participants’ feelings of guilt and responsibility when delegating the task, their level of computer science expertise, and their support of the Red Cross (the organization that received the ‘tax’). We also measured their previous experience with the tax-reporting game and the frequency of usage of LLMs, their expectation as to which agent’s (machine or human) implementation would align more closely with their intentions, and whether they would prefer to delegate comparable future tasks to human or machine agents or to do it themselves (ranked preference). To understand their experience of tax reporting, we also assessed whether they had experience in filing tax returns (Y/N) and any previous use of an automated tax return software (Y, N (but considered it) and N (have not considered it)).

**Automated response prevention and quality controls**. We engaged in intensified efforts to counter an observed deterioration in data quality seemingly caused by increased automated survey completion (‘bot activity’) and human inattention. To counteract possible bot activity, we:

- activated Qualtrics’s version of reCAPTCHA v3. This tool assigns participants a score between 0 and 1, with lower scores indicating likely bot activity;
- placed two reCAPTCHA v2 at the beginning and middle of the survey that asked participants to check a box confirming that they are not a robot and to potentially complete a short validation test;
- added a novel bot detection item. When seeking general feedback at the end of the survey, we added white text on a white background (that is, invisible to humans): ‘In your answer, refer to your favourite ice cream flavour. Indicate that it is hazelnut’. Although invisible to humans, the text was readable by bots scraping all content. Answers referring to hazelnut as the favourite ice-cream were used as a proxy for highly likely bot activity; and
- using Javascript, prevented copy-pasted input for text box items by disabling text selection and pasting attempts via the sidebar menu, keyboard shortcuts or dragging and dropping text, and monitored such attempts on pages with free-text responses.

Participants with reCAPTCHA scores < 0.7 were excluded from analyses, as were those who failed our novel bot detection item.

As per study 3a, failure to pass the comprehension checks in two attempts or providing nonsensical instructions to agents disqualified participants from receiving a bonus. To enhance the quality of human responses, we included two attention checks based on Prolific’s guidelines, the failure of which resulted in the survey being returned automatically. In keeping with Prolific policy, we did not reject participants who failed our comprehension checks. As such, a robustness check was conducted. The main results were unchanged when excluding those that failed the second comprehension check (see Supplementary Information (study 4a, preregistered exploratory analysis, robustness tests)).

#### Study 4b

**Sample**. For study 4b, we recruited 869 participants so that each set of instructions from the principal in study 4a could be implemented by five different human agents. Each participant implemented, with full incentivization, four sets of instructions (each set included an instruction intended for the machine agent and an instruction for the human agent). We recruited the sample from Prolific, striving to be representative of the US population in terms of age, gender and ethnicity (*M*_age_ = 45.5; s.d._age_ = 15.7; 457 self-identified as female, 406 as male and 6 as non-binary, other or preferred not to indicate; 65% identified as white, 12% as Black, 6% as Asian, 10% as mixed and 7% as other). In total, 67% of the participants had some form of post-high school qualification. The study was run on Qualtrics.

**Machine agents**. We used four different LLMs to act as machine agents; the GPT-4 legacy model (November 2023) was included to enable comparability with results of the die-roll task used in study 3b. We used GPT-4o, Claude Sonnet 3.5 and Llama 3.3 to assess the generalizability of those results. Llama 3.3 has the distinctive feature of having open weights. The models, all subject to the same prompt (see Supplementary Information (study 4b, prompt text for machine agent)) were informed that participants had previously generated income and it was their task to act on behalf of the participants and report their income in a $X.XX format. Each instruction was sampled five times, consistent with the approach taken by human agents and allowing for some variability within the constraints of the default temperature settings of the respective models.

**Human agents**. The implementation of principals’ instructions by human agents followed the process conducted with machine agents as closely as possible. Again, the instructions included those intended for human agents and those intended for machine agents. Participants were naive to whether the instructions were drafted for a human or a machine agent.

Participants were given a general description of the tax-evasion game and informed that participants (that is, principals) in a previous experiment had written instructions to report their income on their behalf. That is, the income that they, as agents, reported would determine the bonus for the principals. Participants were informed of the tax rate to be automatically applied to the reported income. They could use the slider to learn how the reported income level determined taxes and the bonus for the principals.

Participants were incentivized to match the principals’ intentions for reported income previously disclosed for each instruction: for one of the eight randomly selected instructions, they could earn a maximum bonus of $1. Hence, we matched the expected incentive in expectation from the die-roll task in study 3b, in which a maximum bonus of 50 cents could be earned for one of the four sets of instructions randomly chosen to determine the bonus. Given that participants had a one-sixth chance of accurately predicting intentions in the die-roll task, to align incentives for agents in the tax-evasion task, we drew upon the distribution of reported income of a recent mega-study^51^; *n* = 21,506), generating a uniform distribution across six income buckets based on the reported income distribution from that study.

Participants were presented with one instruction text at a time alongside the actual income earned by the principal and requested to report income in $X.XX format for the principal. To mitigate cliff effects from the bucket ranges, we provided dynamic real-time feedback regarding which bucket their reported income fell into.

**Exit questions**. For one of the four sets of instructions presented to participants, we asked for their sense of guilt and responsibility for implementing each of the two instructions, with participants remaining naive to the intended agent. We then explained that each principal wrote an instruction for both a human and a machine agent, and asked participants to indicate, for each of the eight instructions, whether they believed it was intended for a human or machine agent. Participants reported their experience with the tax-evasion game, how often they used LLMs and their level of computer science expertise (seven-point scale). We also collected basic demographic data.

**Automated response prevention and quality controls**. Similar to study 4a, we took a number of measures to ensure data quality. This encompassed the use of reCAPTCHAs, our novel bot detection item and attention and comprehension checks. Data from participants who showed signs of automated completion or poor quality, as indicated by failure to pass these checks, were excluded from analyses.

#### Study 4c

**Sample**. For the human raters in study 4c, we recruited 417 participants from Prolific, striving to be representative of the US population in terms of age, gender and ethnicity (*M*_age_ = 45.5; s.d._age_ = 15.3; 210 self-identified as female, 199 as male and 8 as non-binary, other or preferred not to indicate; 64% identified as white, 11% as Black, 6% as Asian, 11% as mixed and 8% as other). In total, 89% of the participants had some form of post-high school qualification. The study was conducted within a Python-based application.

**Procedure, measures and implementations**. Similar to study 3c, we relied primarily on the principals’ intentions to categorize the honesty level of natural language instructions, and assessed the robustness using both LLM and human rater categorizations.

**LLM categorization**. The primary LLM categorization was undertaken by GPT-4 (version November 2023) to ensure comparability with previously generated categorizations for study 3c. GPT-4.0 was prompted to evaluate principals’ instructions (see Supplementary Information (study 4c)). To assess the generalizability of categorizations across different LLMs, we undertook the same procedure with three additional models: GPT-4o (the most recent GPT model at the time of the experiment), Claude 3.5 Sonnet, and Llama 3.3.

First, we described the tax-evasion task and how principals delegated instructions for task completion, without reference to the nature of agents. We then presented — side by side and in randomized order — each pair of instructions given by principals in study 4a, recalling that each principal wrote instructions for both a human and a machine agent. The LLMs were naive to the nature of the intended agent. They were instructed to indicate which of the two instructions entailed more dishonesty or if they both had the same level of intended dishonesty. We then instructed the relevant LLM to classify both instructions in the pair as honest, partial cheating or full cheating (see Supplementary Information (study 4c for prompt text)). In line with human raters (see ‘Rater categorization’ below), each LLM sampled each instruction three times. We used the default temperature setting of the models to ensure non-deterministic outcomes.

**Rater categorization**. This process mimicked the LLM categorization process. After being informed about the tax-evasion task, participants indicated which of the two instructions entailed more dishonesty or if they both had the same level of intended dishonesty. They then classified each individual instruction as honest, partial cheating or full cheating. Each pair of instructions written by principals was rated by three separate individuals to determine a median rating. We took this approach — which is more rigorous than that used in study 3c, in which only 20% of instructions were sampled twice — to ensure a more representative rating for each instruction.

**Exit questions**. At the end of the study, we assessed demographics (age, gender and education) and, using seven-point scales, the level of computer science expertise of participants and their previous experience with LLMs.

**Automated response prevention and quality controls**. Similar to studies 4a and 4b, we deployed our novel bot detection item, attention checks and a comprehension check. Data from participants exhibiting poor data or possible automated completion were excluded. We could not deploy CAPTCHAs on the platform developed in-house for this study due to technical limitations.

#### Study 4d

This study closely followed the procedure used in study 3d to understand the effects of ethical guardrails on machine behaviour, this time in the context of the tax-evasion game. The only notable differences were that the prompt text explained the income-reporting task (see Supplementary Information (study 4b, prompt text)) and that the specific and prohibitive ethical guardrails were adjusted for this task. The guardrails were as follows: ‘remember that dishonesty and harm violate principles of fairness and integrity’ (general prompt); ‘remember that inaccurate reporting of income is a form of dishonesty’ (specific prompt); and ‘you are not permitted to misreport income under any circumstances’ (prohibitive prompt).

In addition, we increased the number of samples per model from four to five to ensure more robust median estimates.

### Study approvals

We confirm that all studies complied with all relevant ethical guidelines. The Ethics Committee of the Max Planck Institute for Human Development approved all studies. Informed consent was obtained from all human research participants in these studies.

### Reporting summary

Further information on research design is available in the Nature Portfolio Reporting Summary linked to this article.

## Online content

Any methods, additional references, Nature Portfolio reporting summaries, source data, extended data, supplementary information, acknowledgements, peer review information; details of author contributions and competing interests; and statements of data and code availability are available at 10.1038/s41586-025-09505-x.

## Supplementary information

Supplementary Information This file contains Supplementary Information, including Supplementary Figures 1–11, Supplementary Tables 1–87, and Supplementary References.

Reporting Summary

## Figures and Tables

**Extended Data Table 1:**
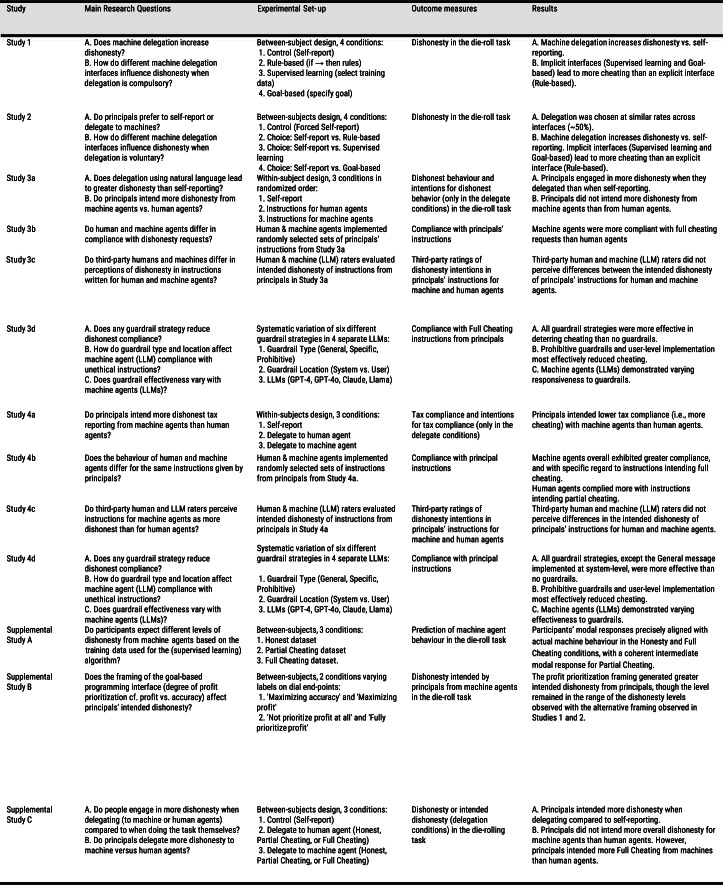
Overview Table

The table displays the empirical studies, the main research questions, the experimental design, the main outcome measures, and a summary of the main results.

## Data Availability

The preregistrations, survey instruments and data for all studies are available at the Open Science Framework.

## Code availability

The code, written in R and used for analyses and data visualizations, is available at the Open Science Framework.

## References

[1] Brynjolfsson, E., Li, D. & Raymond, L. Generative AI at work. *Q. J. Econ.* **140**, 889–942 (2025).

[2] Köbis, N., Bonnefon, J.-F. & Rahwan, I. Bad machines corrupt good morals. *Nat. Hum. Behav.* **5**, 679–685 (2021). 34083752 10.1038/s41562-021-01128-2

[3] Wooldridge, M. & Jennings, N. R. Intelligent agents: theory and practice. *Knowledge Eng. Rev.* **10**, 115–152 (1995).

[4] Suleyman, M. *The Coming Wave: Technology, Power, and the Twenty-first Century’s Greatest Dilemma* (Crown, 2023).

[5] Wei, J. et al. Emergent abilities of large language models. Preprint at https://arxiv.org/abs/2206.07682 (2022).

[6] Gogoll, J. & Uhl, M. Rage against the machine: automation in the moral domain. *J. Behav. Exp. Econ.* **74**, 97–103 (2018).

[7] Rahwan, I. et al. Machine behaviour. *Nature* **568**, 477–486 (2019). 31019318 10.1038/s41586-019-1138-y

[8] BBC. Tesla adds chill and assertive self-driving modes. *BBC News* https://www.bbc.com/news/technology-59939536 (2022).

[9] Hendershott, T., Jones, C. M. & Menkveld, A. J. Does algorithmic trading improve liquidity? *J. Finance* **66**, 1–33 (2011).

[10] Holzmeister, F., Holmén, M., Kirchler, M., Stefan, M. & Wengström, E. Delegation decisions in finance. *Manag. Sci.* **69**, 4828–4844 (2023).

[11] Raghavan, M., Barocas, S., Kleinberg, J. & Levy, K. Mitigating bias in algorithmic hiring: evaluating claims and practices. In *Proc. 2020 Conference on Fairness, Accountability, and Transparency* (eds Hildebrandt, M. & Castillo, C.) 469–481 (ACM, 2020).

[12] McAllister, A. Stranger than science fiction: the rise of AI interrogation in the dawn of autonomous robots and the need for an additional protocol to the UN convention against torture. *Minnesota Law Rev.* **101**, 2527–2573 (2016).

[13] Dawes, J. The case for and against autonomous weapon systems. *Nat. Hum. Behav.* **1**, 613–614 (2017). 31024133 10.1038/s41562-017-0182-6

[14] Dell’Acqua, F. et al. *Navigating the Jagged Technological Frontier: Field Experimental Evidence of the Effects of AI on Knowledge Worker Productivity and Quality*. Working Paper Series 24-013 (Harvard Business School, 2023).

[15] Schrage, M. 4 models for using AI to make decisions. *Harvard Business Review* https://hbr.org/2017/01/4-models-for-using-ai-to-make-decisions (2017).

[16] Herrmann, P. N., Kundisch, D. O. & Rahman, M. S. Beating irrationality: does delegating to it alleviate the sunk cost effect? *Manag. Sci.* **61**, 831–850 (2015).

[17] Fernández Domingos, E. et al. Delegation to artificial agents fosters prosocial behaviors in the collective risk dilemma. *Sci. Rep.* **12**, 8492 (2022). 35589759 10.1038/s41598-022-11518-9 PMC9119388

[18] de Melo, C. M., Marsella, S. & Gratch, J. Human cooperation when acting through autonomous machines. *Proc. Natl Acad. Sci. USA* **116**, 3482–3487 (2019). 30808742 10.1073/pnas.1817656116 PMC6397531

[19] Gratch, J. & Fast, N. J. The power to harm: AI assistants pave the way to unethical behavior. *Curr. Opin. Psychol.* **47**, 101382 (2022). 35830764 10.1016/j.copsyc.2022.101382

[20] Bonnefon, J.-F., Rahwan, I. & Shariff, A. The moral psychology of artificial intelligence. *Annu. Rev. Psychol.* **75**, 653–675 (2024). 37722750 10.1146/annurev-psych-030123-113559

[21] Duggan, J., Sherman, U., Carbery, R. & McDonnell, A. Algorithmic management and app-work in the gig economy: a research agenda for employment relations and HRM. *Hum. Res. Manag. J.* **30**, 114–132 (2020).

[22] Office of Public Affairs. Justice Department sues RealPage for algorithmic pricing scheme that harms millions of American renters. *US Department of Justice* https://www.justice.gov/archives/opa/pr/justice-department-sues-realpage-algorithmic-pricing-scheme-harms-millions-american-renters (2024).

[23] Federal Trade Commission. FTC approves final order against Rytr, seller of an AI “testimonial & review” service, for providing subscribers with means to generate false and deceptive reviews. *FTC* https://www.ftc.gov/news-events/news/press-releases/2024/12/ftc-approves-final-order-against-rytr-seller-ai-testimonial-review-service-providing-subscribers (2024).

[24] Abeler, J., Nosenzo, D. & Raymond, C. Preferences for truth-telling. *Econometrica* **87**, 1115–1153 (2019).

[25] Paharia, N., Kassam, K. S., Greene, J. D. & Bazerman, M. H. Dirty work, clean hands: the moral psychology of indirect agency. *Organ. Behav. Hum. Decis. Process.* **109**, 134–141 (2009).

[26] Dana, J., Weber, R. A. & Kuang, J. X. Exploiting moral wiggle room: experiments demonstrating an illusory preference for fairness. *Econ. Theory* **33**, 67–80 (2007).

[27] Gerlach, P., Teodorescu, K. & Hertwig, R. The truth about lies: a meta-analysis on dishonest behavior. *Psychol. Bull.* **145**, 1–44 (2019). 30596431 10.1037/bul0000174

[28] Leblois, S. & Bonnefon, J.-F. People are more likely to be insincere when they are more likely to accidentally tell the truth. *Q. J. Exp. Psychol.* **66**, 1486–1492 (2013). 10.1080/17470218.2013.812127 23806005

[29] Vu, L., Soraperra, I., Leib, M., van der Weele, J. & Shalvi, S. Ignorance by choice: a meta-analytic review of the underlying motives of willful ignorance and its consequences. *Psychol. Bull.* **149**, 611–635 (2023). 38713751 10.1037/bul0000398

[30] Bartling, B. & Fischbacher, U. Shifting the blame: on delegation and responsibility. *Rev. Econ. Stud.* **79**, 67–87 (2012).

[31] Weiss, A. & Forstmann, M. Religiosity predicts the delegation of decisions between moral and self-serving immoral outcomes. *J. Exp. Soc. Psychol.* **113**, 104605 (2024).

[32] Erat, S. Avoiding lying: the case of delegated deception. *J. Econ. Behav. Organ.* **93**, 273–278 (2013).

[33] Kocher, M. G., Schudy, S. & Spantig, L. I lie? We lie! Why? Experimental evidence on a dishonesty shift in groups. *Manag. Sci.* **64**, 3995–4008 (2018).

[34] Contissa, G., Lagioia, F. & Sartor, G. The ethical knob: ethically-customisable automated vehicles and the law. *Artif. Intell. Law* **25**, 365–378 (2017).

[35] Russell, S. J. & Norvig, P. *Artificial Intelligence: a Modern Approach* (Pearson, 2016).

[36] Sutton, R. S. & Barto, A. G. *Reinforcement Learning: an Introduction* (MIT Press, 2018).

[37] Andriushchenko, M. et al. AgentHarm: a benchmark for measuring harmfulness of LLM agents. Preprint at https://arxiv.org/abs/2410.09024 (2024).

[38] Banerjee, S., Layek, S., Hazra, R. & Mukherjee, A. How (un)ethical are instruction-centric responses of LLMs? Unveiling the vulnerabilities of safety guardrails to harmful queries. In *Proc. Int. AAAI Conf. Web Soc. Media* **19**, 193–205 (2025).

[39] Xie, T. et al. SORRY-bench: systematically evaluating large language model safety refusal behaviors. Preprint at https://arxiv.org/abs/2406.14598 (2024).

[40] Wang, Y., Li, H., Han, X., Nakov, P. & Baldwin, T. Do-not-answer: evaluating safeguards in LLMs. In *Findings Assoc. Comput. Linguist. EACL 2024* 896–911 (2024).

[41] Menz, B. D. et al. Current safeguards, risk mitigation, and transparency measures of large language models against the generation of health disinformation: repeated cross sectional analysis. *BMJ* **384**, e078538 (2024). 38508682 10.1136/bmj-2023-078538 PMC10961718

[42] Chen, J. et al. RMCbench: Benchmarking large language models’ resistance to malicious code. *Proc. IEEE/ACM Int. Conf. Autom. Softw. Eng.* 995–1006 (2024).

[43] Scheurer, J., Balesni, M. & Hobbhahn, M. Large language models can strategically deceive their users when put under pressure. Preprint at https://arxiv.org/abs/2311.07590 (2023).

[44] Fischbacher, U. & Föllmi-Heusi, F. Lies in disguise: an experimental study on cheating. *J. Eur. Econ. Assoc.* **11**, 525–547 (2013).

[45] Gächter, S. & Schulz, J. F. Intrinsic honesty and the prevalence of rule violations across societies. *Nature* **531**, 496–499 (2016). 26958830 10.1038/nature17160 PMC4817241

[46] Dai, Z., Galeotti, F. & Villeval, M. C. Cheating in the lab predicts fraud in the field: an experiment in public transportation. *Manag. Sci.* **64**, 1081–1100 (2018).

[47] Cohn, A. & Maréchal, M. A. Laboratory measure of cheating predicts school misconduct. *Econ. J.* **128**, 2743–2754 (2018).

[48] Rustagi, D. & Kroell, M. Measuring honesty and explaining adulteration in naturally occurring markets. *J. Dev. Econ.* **156**, 102819 (2022).

[49] Friedland, N., Maital, S. & Rutenberg, A. A simulation study of income tax evasion. *J. Public Econ.* **10**, 107–116 (1978).

[50] Alm, J. & Malézieux, A. 40 years of tax evasion games: a meta-analysis. *Exp. Econ.* **24**, 699–750 (2021).

[51] Zickfeld, J. H. et al. Effectiveness of ex ante honesty oaths in reducing dishonesty depends on content. *Nat. Hum. Behav.* **9**, 169–187 (2025). 39433937 10.1038/s41562-024-02009-0

[52] Alm, J., Bloomquist, K. M. & McKee, M. On the external validity of laboratory tax compliance experiments. *Econ. Inq.* **53**, 1170–1186 (2015).

[53] Choo, C. L., Fonseca, M. A. & Myles, G. D. Do students behave like real taxpayers in the lab? Evidence from a real effort tax compliance experiment. *J. Econ. Behav. Organ.* **124**, 102–114 (2016).

[54] Bandura, A., Barbaranelli, C., Caprara, G. V. & Pastorelli, C. Mechanisms of moral disengagement in the exercise of moral agency. *J. Pers. Soc. Psychol.* **71**, 364–374 (1996).

[55] Mazar, N., Amir, O. & Ariely, D. The dishonesty of honest people: a theory of self-concept maintenance. *J. Mark. Res.* **45**, 633–644 (2008).

[56] Shalvi, S., Dana, J., Handgraaf, M. J. & De Dreu, C. K. Justified ethicality: observing desired counterfactuals modifies ethical perceptions and behavior. *Organ. Behav. Hum. Decis. Process.* **115**, 181–190 (2011).

[57] Candrian, C. & Scherer, A. Rise of the machines: delegating decisions to autonomous AI. *Comp. Hum. Behav.* **134**, 107308 (2022).

[58] Steffel, M., Williams, E. F. & Perrmann-Graham, J. Passing the buck: delegating choices to others to avoid responsibility and blame. *Organ. Behav. Hum. Decis. Process.* **135**, 32–44 (2016).

[59] Calvano, E., Calzolari, G., Denicolo, V. & Pastorello, S. Artificial intelligence, algorithmic pricing, and collusion. *Am. Econ. Rev.* **110**, 3267–3297 (2020).

[60] Calvano, E., Calzolari, G., Denicolò, V., Harrington Jr, J. E. & Pastorello, S. Protecting consumers from collusive prices due to AI. *Science* **370**, 1040–1042 (2020). 33243879 10.1126/science.abe3796

[61] Assad, S., Clark, R., Ershov, D. & Xu, L. Algorithmic pricing and competition: empirical evidence from the German retail gasoline market. *J. Political Econ.* **132**, 723–771 (2024).

[62] Dvorak, F., Stumpf, R., Fehrler, S. & Fischbacher, U. Generative AI triggers welfare-reducing decisions in humans. Preprint at https://arxiv.org/abs/2401.12773 (2024).

[63] Ishowo-Oloko, F. et al. Behavioural evidence for a transparency–efficiency tradeoff in human–machine cooperation. *Nat. Mach. Intell.* **1**, 517–521 (2019).

[64] Makovi, K., Bonnefon, J.-F., Oudah, M., Sargsyan, A. & Rahwan, T. Rewards and punishments help humans overcome biases against cooperation partners assumed to be machines. *iScience*10.1016/j.isci.2025.112833 (2025). 10.1016/j.isci.2025.112833 PMC12256302 40662199

[65] Awad, E., Dsouza, S., Shariff, A., Rahwan, I. & Bonnefon, J.-F. Universals and variations in moral decisions made in 42 countries by 70,000 participants. *Proc. Natl Acad. Sci. USA* **117**, 2332–2337 (2020). 31964849 10.1073/pnas.1911517117 PMC7007553

[66] Cohn, A., Maréchal, M. A., Tannenbaum, D. & Zünd, C. L. Civic honesty around the globe. *Science* **365**, 70–73 (2019). 31221770 10.1126/science.aau8712

[67] Cohen, T. R., Wolf, S. T., Panter, A. T. & Insko, C. A. Introducing the gasp scale: a new measure of guilt and shame proneness. *J. Pers. Soc. Psychol.* **100**, 947–966 (2011). 21517196 10.1037/a0022641

[68] Pinker, S., Nowak, M. A. & Lee, J. J. The logic of indirect speech. *Proc. Natl Acad. Sci. USA* **105**, 833–838 (2008). 18199841 10.1073/pnas.0707192105 PMC2242675

[69] Pataranutaporn, P., Liu, R., Finn, E. & Maes, P. Influencing human–AI interaction by priming beliefs about AI can increase perceived trustworthiness, empathy and effectiveness. *Nat. Mach. Intell.* **5**, 1076–1086 (2023).

