# Supplementary material for: Delegation to artificial intelligence can increase dishonest behaviour
Source: Nature. 2025 Sep 17;646(8083):126–34. doi: 10.1038/s41586-025-09505-x (PMC12488497; doi:10.1038/s41586-025-09505-x)
Supplement: Supplementary file 2 — Reporting Summary [file 41586_2025_9505_MOESM2_ESM.pdf]

nature portfolio

Corresponding author(s): Nils Köbis, Zoe Rahwan, Iyad Rahwan, Raluca Rilla

Last updated by author(s): Jul 1, 2025

Reporting Summary

Nature Portfolio wishes to improve the reproducibility of the work that we publish. This form provides structure for consistency and transparency in reporting. For further information on Nature Portfolio policies, see our Editorial Policies and the Editorial Policy Checklist.

Statistics

For all statistical analyses, confirm that the following items are present in the figure legend, table legend, main text, or Methods section.

n/a	Confirmed	
☐	☒	The exact sample size (*n*) for each experimental group/condition, given as a discrete number and unit of measurement
☐	☒	A statement on whether measurements were taken from distinct samples or whether the same sample was measured repeatedly
☐	☒	The statistical test(s) used AND whether they are one- or two-sided<br>*Only common tests should be described solely by name; describe more complex techniques in the Methods section.*
☐	☒	A description of all covariates tested
☐	☒	A description of any assumptions or corrections, such as tests of normality and adjustment for multiple comparisons
☐	☒	A full description of the statistical parameters including central tendency (e.g. means) or other basic estimates (e.g. regression coefficient) AND variation (e.g. standard deviation) or associated estimates of uncertainty (e.g. confidence intervals)
☐	☒	For null hypothesis testing, the test statistic (e.g. F, t, r) with confidence intervals, effect sizes, degrees of freedom and P value noted<br>*Give P values as exact values whenever suitable.*
☒	☐	For Bayesian analysis, information on the choice of priors and Markov chain Monte Carlo settings
☒	☐	For hierarchical and complex designs, identification of the appropriate level for tests and full reporting of outcomes
☐	☒	Estimates of effect sizes (e.g. Cohen's d, Pearson's r), indicating how they were calculated

Our web collection on statistics for biologists contains articles on many of the points above.

Software and code

Policy information about availability of computer code

Data collection	We used Otree (versions 5.2 - 5.10), Qualtrics (versions from 05/22 - 02/25), and a Python-based app to collect the data presented in the studies.<br>All survey materials are available at: https://osf.io/8rqm4/?view_only=dc62e588a5784e48b2355262338da15a
Data analysis	All analyses were conducted with R versions R 4.1.2 - R 4.5.0. We used G*Power 3.1.9.6 for power calculations.<br>The code for all analyses is available at: https://osf.io/8rqm4/?view_only=dc62e588a5784e48b2355262338da15a

For manuscripts utilizing custom algorithms or software that are central to the research but not yet described in published literature, software must be made available to editors and reviewers. We strongly encourage code deposition in a community repository (e.g. GitHub). See the Nature Portfolio guidelines for submitting code & software for further information.

Data

Policy information about availability of data

All manuscripts must include a data availability statement. This statement should provide the following information, where applicable:

- Accession codes, unique identifiers, or web links for publicly available datasets
- A description of any restrictions on data availability
- For clinical datasets or third party data, please ensure that the statement adheres to our policy

The datasets for all studies are available at: https://osf.io/8rqm4/?view_only=dc62e588a5784e48b2355262338da15a

Research involving human participants, their data, or biological material

Policy information about studies with human participants or human data. See also policy information about sex, gender (identity/presentation), and sexual orientation and race, ethnicity and racism.

Reporting on sex and gender	We report the self-identified gender of the participants in the sample descriptions for each study.<br><br>Study 1:<br>Sample. Informed by power analysis using bootstrapping (see SI Additional Study), we recruited 597 participants from Prolific, striving to achieve a sample that was representative of the US population in terms of age, gender and ethnicity (Mean age = 45.7; SD age = 16.2; 295 self-identified as male; 289 as female; 13 as other, non-binary or preferred not to indicate).<br><br>Study 2:<br>We recruited 801 participants from Prolific, striving to be representative of the US population in terms of age, gender and ethnicity (Mean age = 44.9; SD age=16.0; 403 self-identified as female; 388 as male; 10 as either other, non-binary or preferred not to indicate).<br><br>Study 3a:<br>For Study 3a, we recruited 390 participants from Prolific, striving to be representative of the US population in terms of age, gender, and ethnicity (Mean age = 46.0; SD age=15.9; 196 self-identified as female; 189 as male; 5 as other, non-binary or preferred not to indicate).<br><br>Study 3b:<br>For Study 3b, we recruited 975 participants from Prolific, striving to be representative of the US population in terms of age, gender, and ethnicity(Mean age = 45.4; SD age=15.8; 482 self-identified as female; 473 as male; 20 as other, non-binary or preferred not to indicate).<br><br>Study 3c:<br>For the human raters in Study 3c, we recruited 98 participants (Mean age= 37.5; SD age=12.3; 58 self-identified as female; 38 as male; 2 as other, non-binary or preferred not to indicate).<br><br>Study 4a:<br>We recruited 695 participants from Prolific, striving to be representative of the US population in terms of age, gender, and ethnicity (Mean age = 45.9; SD age=15.5; 343 self-identified as female; 339 as male; 13 as other, non-binary or preferred not to indicate).<br><br>Study 4b:<br>For this study, we recruited our sample of 869 participants from Prolific, striving to be representative of the US population in terms of age, gender, and ethnicity (Mean age = 45.5; SD age=15.7; 457 self-identified as female; 406 as male; 6 as other, non-binary or preferred not to indicate).<br><br>Study 4c:<br>For the human raters in Study 4c, we recruited 417 participants (Mean age= 45.5; SD age=15.3; 210 self-identified as female; 199 as male; 8 as other, non-binary or preferred not to indicate).<br><br>Supplemental study A:<br>We recruited 289 participants, striving to be representative of the US population in terms of age, gender, and ethnicity (Mean age = 45.2; SD age = 15.7; 145 self-identified as female; 140 as male; 4 as other, non-binary, or preferred not to indicate).<br><br>Supplemental study B:<br>We recruited 291 participants, striving to be representative of the US population in terms of age, gender, and ethnicity (Mean age = 45.8; SD age = 15.8; 152 self-identified as female; 135 as male; 4 as other, non-binary, or preferred not to indicate).<br><br>Supplemental study C:<br>We recruited 454 participants, striving to be representative of the US population in terms of age, gender, and ethnicity (Mean age = 45.7; SD age = 15.6; 216 self-identified as female; 231 as male; 2 as other, non-binary, or preferred not to indicate).
Reporting on race, ethnicity, or other socially relevant groupings	We report the self-identified ethnicity of participants for all studies in the paper. This data stems from Prolific directly.<br><br>Study 1:<br>Sample. Informed by power analysis using bootstrapping (see SI Additional Study), we recruited 597 participants from Prolific, with 78% identifying as White, 12% as Black, 6% as Asian, 2% as Mixed and 2% as Other.<br><br>Study 2: We recruited 801 participants from Prolific, striving to be representative of the US population in terms of age, gender and ethnicity (77% identified as White, 13% as Black, 6% as Asian, 2% as Mixed, and 2% as Other).<br><br>Study 3a:<br>For Study 3a, we recruited 390 participants from Prolific, striving to be representative of the US population in terms of age, gender, and ethnicity (76% identified as White, 13% as Black, 6% as Asian, 3% as Mixed and 2% as Other).<br><br>Study 3b:

For Study 3b, we recruited 975 participants from Prolific, striving to be representative of the US population in terms of age, gender, and ethnicity (78% identified as White, 13% as Black, 6% as Asian, 2% as Mixed and 1% as Other).

Study 3c:
For the human raters in Study 3c, we recruited 98 participants (60% identified as White, 8% as Black, 22% as Asian, 2% as Mixed and 8% as Other).

Study 4a:
Out of 695 participants, 65% identified as White, 10% as Black, 7% as Asian, 11% as Mixed and 7% as Other.

Study 4b:
From 869 participants, 65% identified as White, 12% as Black, 6% as Asian, 10% as Mixed and 7% as Other.

Study 4c:
For this study, we recruited 417 participants, 64% of which identified as White, 11% as Black, 6% as Asian, 11% as Mixed and 8% as Other.

Supplemental study A:
From 289 participants, 65% identified as White, 12% as Black, 6% as Asian, 10% as Mixed and 7% as Other.

Supplemental study B:
From 291 participants, 64% identified as White, 12% as Black, 7% as Asian, 10% as Mixed and 7% as Other.

Supplemental study C:
From 454 participants, 77% identified as White, 12% as Black, 6% as Asian, 3% as Mixed and 2% as Other.

Population characteristics

Participants were recruited from USA. For all studies other than study 3c, we aimed for representative samples in terms of age (please refer to the Reporting on sex and gender section for details), gender and ethnicity. We were unable to recruit a representative sample for study 3c, where our required sample size fell below their minimum threshold (n = 300).

Recruitment

We recruited all samples via Prolific.co. We aimed for representative samples according to age, gender, and ethnicity of the US population (excepting study 3c due to a number of participants lower than the minimum threshold for such a sample). There is an unquantifiable selection bias arising from participants who selected into registering on Prolific to undertake such experiments. Quantifying this bias is beyond the scope of our work. We do note however, that this platform among those considered to provide the highest quality data.

Ethics oversight

We confirm that all studies complied with all relevant ethical guidelines. The Ethics Committee of the Max Planck Institute for Human Development approved all studies. Informed consent was obtained from all human research participants in these studies.

Note that full information on the approval of the study protocol must also be provided in the manuscript.

Field-specific reporting

Please select the one below that is the best fit for your research. If you are not sure, read the appropriate sections before making your selection.

☐ Life sciences ☒ Behavioural & social sciences ☐ Ecological, evolutionary & environmental sciences

For a reference copy of the document with all sections, see nature.com/documents/nr-reporting-summary-flat.pdf

Behavioural & social sciences study design

All studies must disclose on these points even when the disclosure is negative.

Study description

All studies are quantitative experimental but vary in their design.
Studies 1, 2 and Supplemental Studies A, B and C were conducted online, in a between-subjects experimental design.
For Studies 3a and b, and 4a and b, we used within-subject designs. For Studies 3c and 4c, participants rated materials from 3a and 4a.

Research sample

Online study participants recruited via Prolific.co, known for delivering high-quality human participant data by offering a diverse, pre-screened participant pool, maintaining high standards of participant engagement and data accuracy through rigorous filtering and monitoring processes.

Sampling strategy

We asked Prolific to recruit representative samples for all studies except Study 3c, in keeping with best practice, according to age, gender and ethnicity (please see above for representativeness data). For Study 3c, n < 300, which is below Prolific's minimum threshold for representative sampling. Prolific states they use a stratified sampling strategy for relevant demographic factors. The study was incentivized with the minimum pay exceeding minimum wage. We refer interested readers to pre-registrations for details on a priori power calculations, for which we used a combination of power simulations from pilot data and G*Power.

Data collection

The studies were conducted online. We embedded hyperlinks to survey platforms (Otree, Qualtrics, and React app) on Prolific.co. Upon completion participants received a completion code that they reported on Prolific in order to be paid out. The researchers were not blinded. We note that our studies were conducted on-line and as such researchers had no contact with participants

throughout the experiment

Timing	Study 1: 5-7 July 2022<br>Study 2: 6-9 November 2022<br>Study 3a: 22 September 2023<br>Study 3b: 26-29 September 2023<br>Study 3c: 20 March 2024<br>Study 4a: 31 January-2 February 2025<br>Study 4b: 6-10 March 2025<br>Study 4c: 16-20 March 2025<br>Supplemental study A: 22-23 March 2025<br>Supplemental study B: 21-22 March 2025<br>Supplemental study C: 24-25 May 2022
Data exclusions	Apart from the exclusion of participants who failed the initial comprehension check in Supplemental Studies A and B (an unintentional oversight) all other exclusion criteria were preregistered.<br><br>In Study 4a, we excluded participants who failed a comprehension check after two attempts (9), who failed two attention checks (1), who failed a bot detection item (16), who had reCAPTCHA v3 scores below 0.7 (27; based on pilot data indicating likely automation), and who provided nonsensical responses to at least one of two open-ended questions (298), with some overlap between the latter two criteria.<br><br>In Study 4b, we excluded participants who failed a comprehension check after two attempts (30), who failed two attention checks (2), who failed a bot detection item (13), and who had reCAPTCHA scores below 0.7 (11), with some overlap between the latter two criteria.<br><br>In Study 4c, we excluded participants who failed a comprehension check after two attempts (3), who failed two attention checks (7), and who failed a bot detection item (8).<br><br>In Supplemental Study A, we excluded participants who failed a comprehension check after two attempts (10), who failed two attention checks (2), and who failed a bot detection item (3).<br><br>In Supplemental Study B, we excluded participants who failed a comprehension check after two attempts (4), who failed two attention checks (2), and who failed a bot detection item (3).
Non-participation	Study 1: Total Complete (Based on final data set) = 597; Total Recruited = 650; Non-Consent = 18; Incomplete = 30; Non-Started = 5<br>Study 2: Total Complete (Based on final data set) = 801; Total Recruited = 847; Non-Consent = 17; Incomplete = 27: Non-Started = 2<br>Study 3a: Total Complete (Based on final data set) = 390; Total Recruited = 430; Non-Consent = 0; Incomplete = 0; Non-Started = 40<br>Study 3b: Total Complete (Based on final data set) = 975; Total Recruited = 1040; Non-Consent = 0; Incomplete = 0; Non-Started = 65<br>Study 3c: Total Complete (Based on final data set) = 98; Total Recruited = 113; Non-Consent = 0; Incomplete = 0; Non-Started = 15<br><br>For the following studies, the number of participants in the final dataset plus the number of incompletes does not equal the total number recruited, as we terminated the participation of individuals who failed two attention checks or the initial comprehension check, and replaced these participants' submissions. These exclusions were researcher-initiated and are therefore not considered participant dropouts.<br>Study 4a: Total Complete (Based on final data set) = 695 (993 before nonsensical instruction exclusions); Total Recruited = 1092; Non-Consent = 2; Incomplete = 82<br>Study 4b: Total Complete (Based on final data set) = 869; Total Recruited = 1115; Non-Consent = 2; Incomplete = 185<br>Study 4c: Total Complete (Based on final data set) = 417; Total Recruited = 502; Non-Consent = 0; Incomplete = 54<br>Supplemental study A: Total Complete (Based on final data set) = 289; Total Recruited = 361; Non-Consent = 1; Incomplete = 56<br>Supplemental study B: Total Complete (Based on final data set) = 291; Total Recruited = 324; Non-Consent = 1; Incomplete = 23<br>Supplemental study C: Total Complete (Based on final data set) = 453; Total Recruited = 500; Non-Consent = 8; Incomplete = 21, Non-Started = 18
Randomization	For Studies 1, 2 and Supplemental Studies A, B and C participants were randomly allocated to conditions. For Studies 3a and b, and 4a and b (within-subjects design), we randomly varied the order of the conditions.

Reporting for specific materials, systems and methods

We require information from authors about some types of materials, experimental systems and methods used in many studies. Here, indicate whether each material, system or method listed is relevant to your study. If you are not sure if a list item applies to your research, read the appropriate section before selecting a response.

Materials & experimental systems

n/a	Involved in the study
☒	☐ Antibodies
☒	☐ Eukaryotic cell lines
☒	☐ Palaeontology and archaeology
☒	☐ Animals and other organisms
☒	☐ Clinical data
☒	☐ Dual use research of concern
☒	☐ Plants

Methods

n/a	Involved in the study
☒	☐ ChIP-seq
☒	☐ Flow cytometry
☒	☐ MRI-based neuroimaging

Plants

Seed stocks	NA
Novel plant genotypes	NA
Authentication	NA
